# Aidi injection, a traditional Chinese biomedical preparation for gynecologic tumors: a systematic review and PRISMA-compliant meta-analysis

**DOI:** 10.1042/BSR20204457

**Published:** 2021-03-05

**Authors:** Xue Li, Chengming Xiao, Kai Qu

**Affiliations:** 1 Department of Obstetrics and Gynecology, Liaocheng People’s Hospital, Liaocheng 252000, Shandong Province, P.R. China; 2 Department of Hepatobiliary Surgery, The First Affiliated Hospital of Xi’an Jiaotong University, Xi’an 710061, Shaanxi Province, P.R. China

**Keywords:** Aidi injection, conventional treatment, gynecologic tumor, meta-analysis, traditional Chinese biomedical preparation

## Abstract

Aidi injection (ADI), a traditional Chinese biomedical preparation, is a promising adjuvant therapy for gynecologic tumors (GTs), including cervical cancer (CC), endometrial cancer (EC), and ovarian cancer (OC). Although studies have reported positively on ADI therapy, its exact effects and safety in GT patients remain controversial. Therefore, a wide-ranging systematic search of electronic databases was performed for this meta-analysis. Data from 38 trials including 3309 GT patients were analyzed. The results indicated that the combination of conventional treatment and ADI markedly improved the patients’ overall response rate (*P*<0.00001), disease control rate (*P*<0.00001), and quality of life (*P*<0.05) compared with conventional treatment alone. Furthermore, patient immunity was enhanced with combined treatment, as indicated by significantly increased percentages of CD3^+^ (*P*=0.005) and CD4^+^ (*P*<0.00001) and increased CD4^+^/CD8^+^ ratio (*P*=0.001). Most of the adverse events caused by radiochemotherapy such as gastrointestinal issues, leukopenia, thrombocytopenia, and hepatotoxicity, (*P*<0.05 for all) were significantly alleviated when ADI was used in the GT patients. However, other adverse events such as nephrotoxicity, diarrhea, alopecia, and neurotoxicity did not significantly differ between the two groups. Overall, these results suggest that the combination of conventional and ADI treatment is more effective than conventional treatment alone.

## Introduction

Gynecologic tumors (GTs) pose a serious threat to the health and well-being of women, as they are the leading causes of cancer-related death worldwide. GTs mainly comprises cervical cancer (CC), endometrial cancer (EC), and ovarian cancer (OC), which are the 10th, 17th, and 20th most common cancers, respectively [1,2]. In 2018, approximately 1,247,330 newly diagnosed GT cases and 586,093 GT-related deaths occurred worldwide [1,2]. GT treatment includes different management strategies such as surgery, radiotherapy, and chemotherapy [3–6]. Although these therapeutic methods have greatly advanced in the past few decades, the prognosis of GT remains poor, as they are mostly diagnosed at stages III or IV [3–10]. In individuals with extensive invasion and distant metastasis, the management of these tumors is typically aimed at enhancing the quality of life (QoL) and survival rate, because current conventional treatments cannot be used to completely remove the tumor [3–6,8–11]. Moreover, the unpleasant side effects of GT treatment are one of the most important factors limiting the clinical application of radiochemotherapy.

Recently, traditional Chinese medicine has been widely used as an auxiliary treatment for malignancies, with promising therapeutic effects reported by several clinical studies [12–17]. Aidi injection (ADI) is an important injectable prepared from the extracts of various Chinese herbs: Mylabris phalerata, Radix astragali (*Astragalus membranaceus* [Fisch.] Bge. root), Radix ginseng (*Panax ginseng* C.A. Meyer root), and Acanthopanax senticosus (*Acanthopanax senticosus* [Rupr. & Maxim.] Harms) [18–20]. A study on the chemical constituents of ADI reported that 22 chemical components were detected and isolated from the preparation [19,21]. The main active ingredients included cantharidin, cantharidate, astragaloside, ginsenoside, elentheroside E, isofraxidin, syringin coniferin, among others [18,19]. ADI has been approved by the Chinese State Food and Drug Administration (SFDA) for the treatment of various malignant tumors when used alone or in combination with other drugs [19]. Previous studies have suggested that ADI mediates anti-tumoral effects by improving the body’s immunity, inducing tumor cell apoptosis, and inhibiting tumor cell proliferation [18–23]. It can also significantly improve the efficacy of radiochemotherapy and reduce any associated adverse events [18–20].

**Figure 1:**
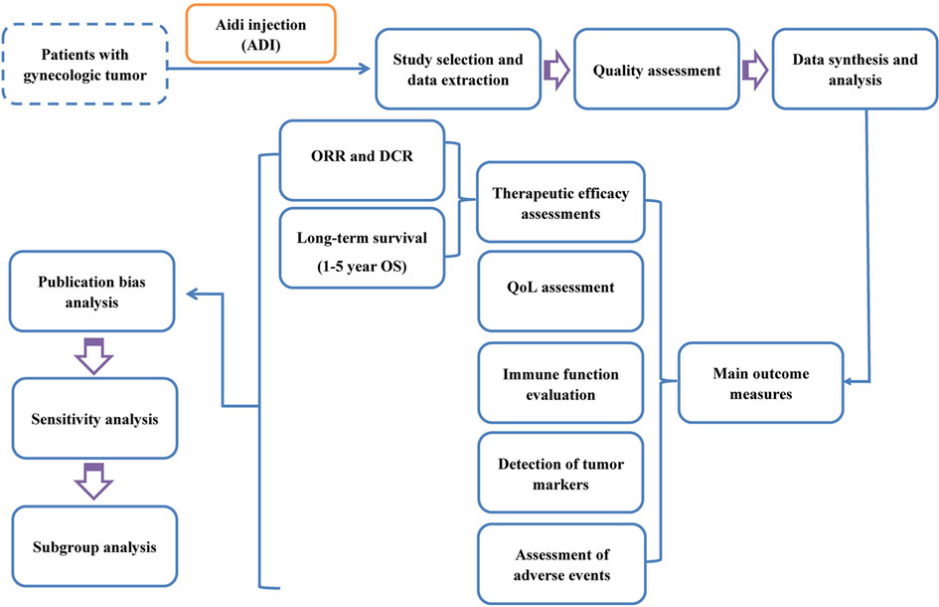
Work flow of the present study

Several clinical studies have suggested that GT patients may benefit from ADI-mediated therapy [24–61]. However, despite extensive studies, the clinical efficacy and safety of conventional treatment combined with ADI have not been systematically evaluated. In the present study, we conducted a meta-analysis to determine the efficacy and safety of conventional GT treatment in combination with ADI compared with conventional GT treatment alone. This may provide insights that can be used for the development of new treatment strategies for GT patients (Figure 1).

## Methods

This meta-analysis was performed in accordance with the Preferred Reporting Items for Systematic Reviews and Meta-Analyses (PRISMA) guidelines [62].

### Search strategy

Eligible prospective controlled clinical trials were searched from the following electronic databases: Web of Science, PubMed, Cochrane Library, EMBASE, Medline, Chinese Biological Medicine Database (CBM), China National Knowledge Infrastructure (CNKI), Chinese Scientific Journal Database (CSJD), and the Wanfang database. Publications in English and Chinese dated from the inception of the database to December 2020 were shortlisted using the following search terms: ‘Aidi injection’ or ‘Ai-di injection’ or ‘Aidi zhusheye’ or ‘ADI’ combined with ‘gynecologic oncology’ or ‘gynecologic tumor’ or ‘gynecologic carcinoma’ or ‘gynecologic cancer’ or ‘ovarian oncology’ or ‘ovarian tumor’ or ‘ovarian carcinoma’ or ‘ovarian cancer’ or ‘cervical oncology’ or ‘cervical tumor’ or ‘cervical carcinoma’ or ‘cervical cancer’ or ‘endometrial oncology’ or ‘endometrial tumor’ or ‘endometrial carcinoma’ or ‘endometrial cancer’ or ‘EC’ or ‘OC‘ or ‘CC’. No other were restrictive search criteria applied.

### Eligibility criteria

#### Inclusion criteria

- Studies wherein GT had been confirmed using cytological or pathological diagnostic methods (OC, CC, or EC).
- All available randomized controlled trials and high-quality prospective cohort studies involving GT patients.
- Studies involving more than 30 GT patients.
- Studies comparing the clinical outcomes of conventional treatment plus ADI adjuvant therapy (experimental group) with those of conventional treatment alone (control group); conventional treatment comprised surgery, radiation treatment, or chemotherapy.

#### Exclusion criteria

Duplicated studies, publications without sufficient data, noncomparative clinical trials, case reports and series, meta-analyses, literature reviews, meeting abstracts, and other unrelated studies were excluded from the analysis.

### Data extraction and management

Data were independently extracted by two investigators (Li, X. and Xiao, C.M.) using the aforementioned inclusion and exclusion criteria; disagreements were adjudicated by a third reviewer (Qu, K.).

The following data were extracted from eligible studies:

- Study characteristics such as name of the first author, year of publication, and sample size.
- Patient characteristics such as tumor stage and age.
- Details of the interventions such as intervention technique as well as dosage, administration route, and duration of ADI treatment.
- Outcomes measures and other parameters that included the overall response rate (ORR), disease control rate (DCR), overall survival (OS), QoL, immune indexes (CD3^+^, CD4^+^, and CD8^+^ percentages and CD4^+^/CD8^+^ cell ratios), tumor markers (HE4, CA125, CEA, and CA199), and adverse effects.

We attempted to contact the authors to request missing or incomplete data. If the relevant data could not be acquired, the studies were excluded from the analysis.

### Quality assessment

To ensure the quality of the meta-analysis, the quality of the included randomized and nonrandomized controlled trials was evaluated according to the Cochrane Handbook tool [63] and Methodological Index for Nonrandomized Studies (MINRRS, Table 2), respectively [64].

### Types of outcome measures

#### Main outcomes

The primary outcomes for the present analysis included clinical efficacy and adverse effects, as defined by the Response Evaluation Criteria in Solid Tumors 1.1 (RECIST Criteria 1.1) [65].

- Short-term clinical efficacy was defined as the short-term tumor response measured by the ORR (sum of complete and partial response rates) and DCR (sum of complete response, partial response, and stable disease rates).
- Adverse events included gastrointestinal adverse effects, leukopenia, and thrombocytopenia, among others.

#### Secondary outcomes

- Long-term clinical efficacy was determined using 1-, 2-, 3-, and 5-year OS.
- QoL was evaluated using the quality of life improved rate (QIR) and the Karnofsky score (KPS).
- Immune function of the GT patients was assessed using CD3^+^, CD4^+^, and CD8^+^ percentages, and CD4^+^/CD8^+^ ratios.
- Presence of tumor markers, namely HE4, CA125, CEA, and CA199, was evaluated.

### Statistical analysis

Review Manager 5.3 (Nordic Cochran Centre, Copenhagen, Denmark) and Stata 14.0 (Stata Corp., College Station, TX, U.S.A.) statistical software were used for statistical analyses. Heterogeneity of treatment effects across trials was assessed using Cochrane’s *Q* test and *I^2^* statistics [66]. A *P*-value > 0.1 and *I^2^* < 50% suggested that there was no statistical heterogeneity, and the fixed-effects model was used for meta-analysis; otherwise, a random-effects model was used to calculate the outcomes. Continuous data were presented as standardized mean difference with corresponding 95% confidence intervals (CIs). Dichotomous data were reported as odds ratios (OR) with 95% CIs. A two-tailed *P*-value < 0.05 was considered statistically significant. Any publication bias was investigated using funnel plots and the Begg’s and Egger’s tests for parameters that were reported in more than 10 studies [67–69]. A trim-and-fill method was used to coordinate the estimates from unpublished studies if publication bias existed, and the adjusted results were compared with the original pooled OR [70]. Sensitivity analysis was performed to explore an individual study’s influence on the pooled results by deleting one study at a time from the pooled analysis.

## Results

### Search results

**Figure 2:**
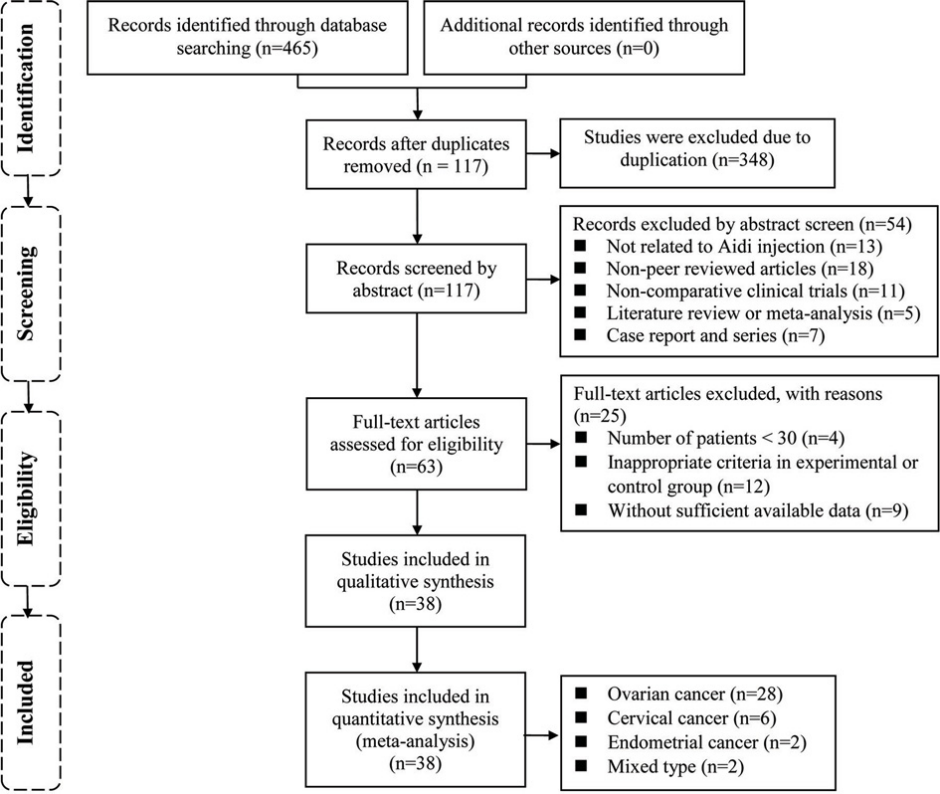
Study selection process for the meta-analysis

The initial search retrieved a total of 465 articles, of which 348 were excluded due to duplication. After the title and abstract review, 44 articles were further excluded for the following reasons: not related to ADI (*n*=13), non-peer reviewed articles (*n*=18), non-comparative clinical trials (*n*=11), literature reviews or meta-analyses (*n*=5), and case reports and series (*n*=7). Thus, 63 studies were potentially eligible. After a detailed assessment of full texts, studies with less than 30 GT patients (*n*=4), trials with inappropriate inclusion and exclusion criteria (*n*=12), and papers with insufficient data (*n*=9) were excluded. Ultimately, 38 trials (OC, *n*=28; CC, *n*=6; EC, *n*=2; and mixed type, *n*=2) [24–61] involving 3309 patients with OC, CC, or EC were included in the final analysis (Figure 2).

### Patient characteristics

All included studies were conducted at different medical centers in China. In total, 1729 GT patients were treated using conventional methods in combination with ADI, whereas 1580 patients were treated using conventional methods alone. The ADI used in all the included studies was manufactured by Guizhou Yibai Pharmaceutical Co., Ltd. with a manufacturing approval number issued by the Chinese SFDA (Z52020236). The study and patient characteristics are summarized in Table 1.

**Table 1:** Clinical information from the eligible trials in the meta-analysis

Included studies	Tumor stage	Tumor stage	Patients Con/Exp	Age (year) Con vs Exp group	Intervening methods	Dosage of Aidi injection	Duration of treatments	Parameter types
Ai, H.L. (2013)	OC (36) CC (14)	Not provided	25/25	56–79 vs 55–83 (range)	CT vs CT+ Aidi injection (ID)	100 ml/time^*^	2 weeks/course, 2 courses.	④
Cao, F.B. (2016)	OC	III-IV	15/15	36–72 vs 32–68 (range)	CT vs CT+ Aidi injection (ID)	Not provided	Not provided	①③④
Cao, Q.X. (2016)	OC	III-IV	35/35	48.76 ± 4.59 vs 50.12 ± 6.36 (mean)	CT vs CT+ Aidi injection (ID)	60 ml/time*	3 weeks/course, 2 courses.	①③④
Chen, T. (2009)	OC	III-IV	29/29	35–71 vs 33–69 (range)	CT vs CT+ Aidi injection (ID)	50 ml/time^*^	3 weeks/course, 2 courses.	①③④
Cheng, H.J. (2006)	CC	II-IV	40/48	24–82 (range)	CT vs CT+ Aidi injection (ID)	50 ml/time^*^	3-4 weeks/course, 1 course.	①②③④
Cui, Y.Y. (2017)	OC	Not provided	39/39	57.37 ± 7.12 vs 57.41 ± 7.03 (mean)	CT vs CT+ Aidi injection (ID)	70 ml/time^*^	3 weeks/course, 2 courses.	①⑤⑥
Deng, L. (2007)	OC (27) CC (22) EC (13)	III-IV	30/32	29–71 vs 31–73 (range)	CT vs CT+ Aidi injection (ID)	50 ml/time^*^	15 days/course, 2 courses.	①④
Fu, J.H. (2013)	OC	II-IV	47/49	63 ± 4.2 vs 64 ± 3.5 (mean)	CT vs CT+ Aidi injection (ID)	80 ml/time^*^	3 weeks/course, 3 courses.	②③④
Hu, W. (2014)	OC	III-IV	32/40	32–70 vs 42–74 (range)	CT vs CT+ Aidi injection (ID)	50–100 ml/time^*^	3 weeks/course, 2 courses.	①③④
Hu, Y.F. (2011)	CC	II-IV	34/73	45–75 (range)	CT vs CT+ Aidi injection (ID)	50 or 100 ml/time^*^	2 weeks/course, 3 courses.	①③
Huang, L.J. (2018)	EC	III-IV	34/34	52.1 ± 7.0 vs 51.4 ± 7.6 (mean)	CT vs CT+ Aidi injection (ID)	50 ml/time*	3 weeks/course, 3 courses.	①③④⑤⑥
Jiang, L. (2011)	EC	I	28/30	52.1 ± 7.0 vs 51.4 ± 7.6 (mean)	CT vs CT+ Aidi injection (ID)	100 ml/time^*^	10days	③④
Lan, G.H. (2017)	OC	III-IV	29/29	50.0 ± 1.2 vs 47.0 ± 2.5 (mean)	CT vs CT+ Aidi injection (ID)	50 ml/time*	3 weeks/course, 2 course.	①③
Lan, S.L. (2013)	OC	III-IV	28/30	43–75 vs 41–76 (range)	CT vs CT+ Aidi injection (ID)	100 ml/time^*^	10 days/course, 2-4 courses.	①③④
Lan, Y.L. (2011)	OC	III-IV	26/26	48.2 ± 5.6 (mean)	CT vs CT+ Aidi injection (ID)	50 ml/time*	3 weeks/course, 2 course.	①④
Li, Y.F. (2007)	OC	III-IV	20/21	27–74 (range)	CT vs CT+ Aidi injection (ID)	50 ml/time^*^	Not provided	①③
Li, Z.W. (2012)	OC	Not provided	30/30	40–67 vs 42–65 (range)	CT vs CT+ Aidi injection (ID)	50 ml/time^*^	3 weeks/course, 2 courses.	①④
Liu, J. (2015)	OC	III-IV	36/36	48.1 ± 7.5 vs 45.4 ± 6.8 (mean)	CT vs CT+ Aidi injection (ID)	50 ml/time^*^	3 weeks/course, 2 courses.	①③④
Liu, T. (2008)	OC	III-IV	40/40	31–72 (range)	CT vs CT+ Aidi injection (ID)	60 ml/ time^*^	3 weeks/course, 2-6 courses.	①③④
Lu, L. (2016)	OC	III-IV	40/40	46.8 ± 3.9 vs 46.5 ± 3.7 (mean)	CT vs CT+ Aidi injection (ID)	80 ml/time*	3 weeks/course, 2 courses.	①③④
Lv, J. (2003)	OC	III-IV	32/35	31–72 vs 34–74 (range)	CT vs CT+ Aidi injection (ID)	50 ml/time^*^	20 days/course, 2-3 courses.	①③
Ma, Y. (2009)	OC	III-IV	27/31	30–79 (range)	CT vs CT+ Aidi injection (ID)	100 ml/time^*^	10 days/course, 2 courses.	①③④⑤
Ma, Y.Q. (2016)	CC	II-III	44/60	54 ± 10.77 vs 52 ± 9.78 (mean)	CT vs CT+ Aidi injection (ID)	80 ml/time*	3 weeks/course	①③④⑤
Nian, L. (2019)	OC	III-IV	75/75	41.1 ± 3.9 vs 42.3 ± 4.7 (mean)	CT vs CT+ Aidi injection (ID)	50 ml/time^*^	4 weeks/course, 2 courses.	①③
Pu, S.J. (2015)	OC	Not provided	250/250	56.42 ± 2.03 vs 57.28 ± 2.17 (mean)	CT vs CT+ Aidi injection (ID)	80 ml/time^*^	3 weeks/course, 2 courses.	③④
Qi, M.G. (2012)	OC	III-IV	40/40	54.1 ± 10.4 vs 53.4 ± 10.2 (mean)	CT vs CT+ Aidi injection (ID)	50 ml/time^*^	2 weeks	②
Shao, B. (2019)	OC	III-IV	25/25	47.23 ± 6.89 vs 48.51 ± 7.12 (mean)	CT vs CT+ Aidi injection (ID)	50 ml/time^*^	3 weeks/course, 2 courses.	①④⑤
Song, J.W. (2013)	OC	Not provided	46/99	Not provided	CT vs CT+ Aidi injection (ID)	40–60 ml/time^*^	Not provided	①
Wang, Y.F. (2006)	OC	III-IV	45/48	38–73 (range)	CT vs CT+ Aidi injection (ID)	50 ml/time*	15 days	①③
Wei, M. (2014)	OC	III-IV	36/41	48.9 ± 6.5 vs 58.3 ± 6.2 (mean)	CT vs CT+ Aidi injection (ID)	Not provided	Not provided	②
Wei, X.S. (2018)	OC	III-IV	35/35	48.75 ± 4.58 vs 50.11 ± 6.35 (mean)	CT vs CT+ Aidi injection (ID)	60 ml/time^*^	3 weeks/course, 2 courses.	①④
Yu, J. (2015)	CC	II-IV	60/60	42.9 ± 3.3 vs 43.7 ± 3.5 (mean)	CT vs CT+ Aidi injection (ID)	80 ml/time^*^	4-6 weeks	①③
Zhang, H.Y. (2019)	OC	III-IV	45/45	53.43 ± 2.22 vs 53.71 ± 2.53 (mean)	CT vs CT+ Aidi injection (ID)	80 ml/time^*^	3 weeks/course, 2 courses.	①③④⑤
Zhang, T.F. (2017)	CC	Not provided	59/59	42.6 ± 3.8 vs 43.5 ± 3.7 (mean)	CT vs CT+ Aidi injection (ID)	50–100 ml/time^*^	4 weeks/course, 2-4 courses.	①④
Zhong, R.Z. (2014)	OC	III-IV	26/26	40.9 ± 7.7 vs 41.2 ± 7.2 (mean)	CT vs CT+ Aidi injection (ID)	50 ml/time*	30 days/course, 2 courses.	①③④
Zhou, M. (2018)	OC	III-IV	44/45	42.5 ± 1.3 vs 42.2 ± 1.5 (mean)	CT vs CT+ Aidi injection (ID)	50 ml/time^*^	3 weeks/course, 2 courses.	①③④
Zhou, Y.Q. (2011)	CC	II-IV	28/28	35–66 (range)	CT vs CT+ Aidi injection (ID)	80 ml/time^*^	2 weeks/course, 2 courses.	①③④
Zhu, Y.H. (2014)	OC	III-IV	26/26	47.61 ± 3.81 vs 47.36 ± 3.97 (mean)	CT vs CT+ Aidi injection (ID)	80 ml/time^*^	3 weeks/course, 2 courses.	④

**Notes:** Control group: conventional treatments alone group; Experimental group: conventional treatments and Aidi injection combined group. ①: Overall response rate and/or Disease control rate; ②: Overall survival; ③: adverse events; ④: quality of life; ⑤: Immune function index; ⑥: Tumor markers.

* : 1 time/day.

Abbreviations**:** CC, cervical carcinoma; CT, conventional treatments; EC, endometrial cancer; ID, Intravenous drip; OC, ovarian cancer.

### Quality assessment

**Figure 3:**
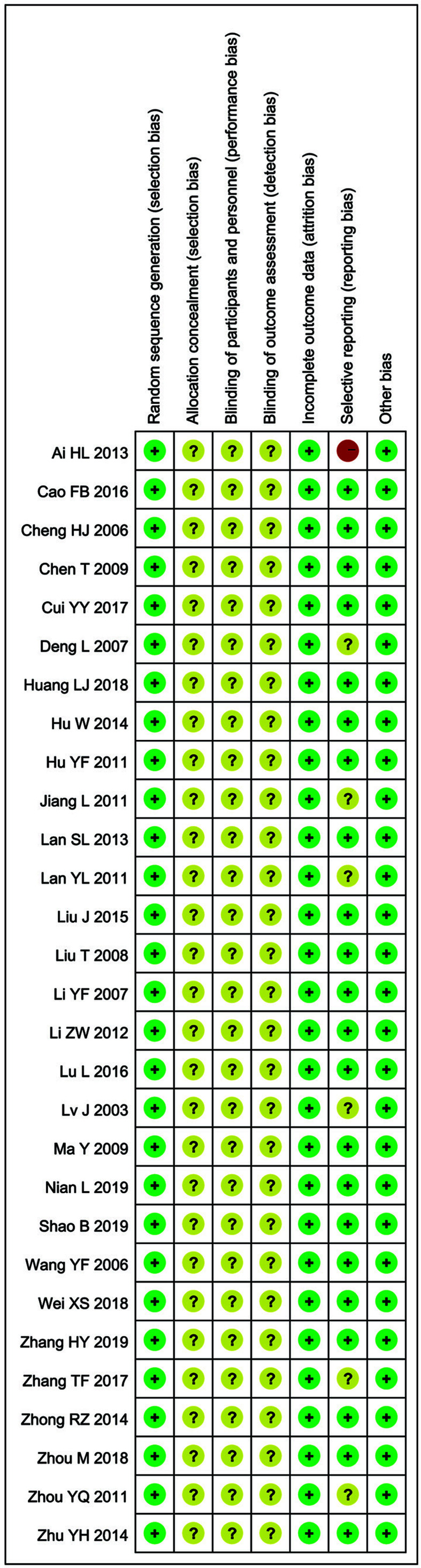
Risk of bias summary Review of authors’ judgments about each risk of bias item for included studies. Note: Each color represents a different level of bias: red for high-risk, green for low-risk, and yellow for unclear-risk of bias.

Quality assessment of the risk of bias has been given in Figure 3 and Table 2. The results revealed that the literature retrieved for the present study was of average quality.

**Table 2:** Quality assessment of nonrandomized comparative studies

Study	Nonrandomized studies								Additional criteria in comparative study				Total
	A	B	C	D	E	F	G	H	I	J	K	L	
Cao, Q.X. (2016)	2	1	2	2	1	1	2	0	2	2	2	2	19
Fu, J.H. (2013)	2	1	2	2	1	2	1	0	2	2	2	2	19
Lan, G.H. (2017)	2	1	2	2	1	1	2	0	2	2	2	2	19
Ma, Y.Q. (2016)	2	1	2	2	1	1	2	0	2	2	2	2	19
Pu, S.J. (2015)	2	1	2	1	1	1	2	0	2	2	2	2	18
Qi, M.G. (2012)	2	1	2	1	1	2	2	0	2	2	2	2	19
Song, J.W. (2013)	2	1	2	1	1	1	2	0	2	2	2	2	18
Wei, M. (2014)	2	1	2	1	1	2	2	0	2	2	2	2	19
Yu, J. (2015)	2	1	2	2	1	1	2	0	2	2	2	2	19

A: A clearly stated aim; B: Inclusion of consecutive patients; C: Prospective collection of data; D: Endpoints appropriate to the aim of the study; E: Unbiased assessment of the study endpoint; F: Follow-up period appropriate to the aim of the study; G: Loss to follow-up less than 5%; H: Prospective calculation of the study size; I: An adequate control group; J: Contemporary groups; K: Baseline equivalence of groups; L: Adequate statistical analyses.

**Notes:** The items are scored 0 (not reported), 1 (reported but inadequate), and 2 (reported and adequate).

### Assessment of therapeutic efficacy

#### ORR and DCR

**Figure 4:**
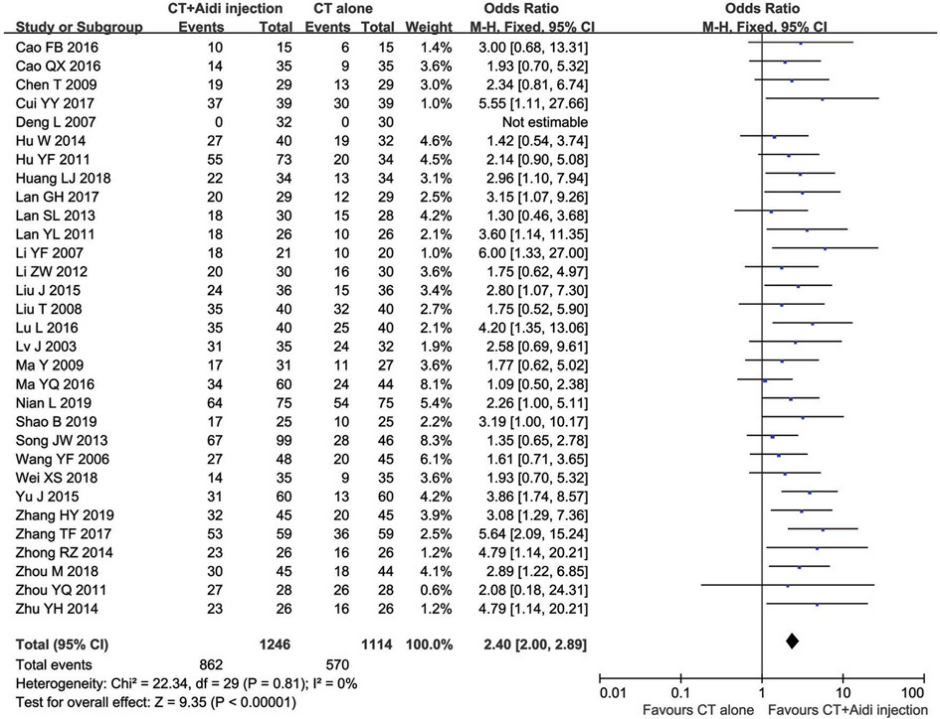
Forest plot of overall response rate in patients treated with CT+Aidi injection and CT alone CT, Conventional treatment. The fixed effects meta-analysis model (Mantel–Haenszel method) was used.

**Figure 5:**
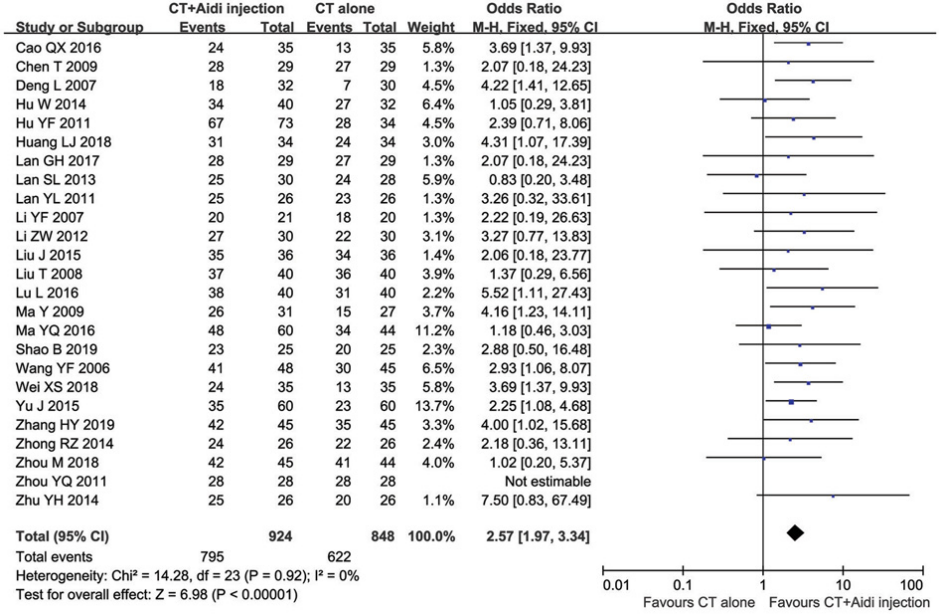
Forest plot of disease control rate in patients treated with CT+Aidi injection and CT alone CT, Conventional treatment. The fixed effects meta-analysis model (Mantel–Haenszel method) was used.

Thirty-one clinical trials [25–27,29,30,32–47,50–52,54–61], involving 2360 patients, compared the ORR and/or DCR between the two groups. The pooled results revealed that patients who underwent combination therapy had improved ORR (Figure 4; OR = 2.40, 95% CI = 2.00–2.89, *P*<0.00001) and DCR (Figure 5; OR = 2.57, 95% CI = 1.97–3.34, *P*<0.00001), compared with those who received conventional treatments alone. Fixed-effect models were used to analyze OR rate because of low heterogeneity.

#### Long-term survival

**Figure 6:**
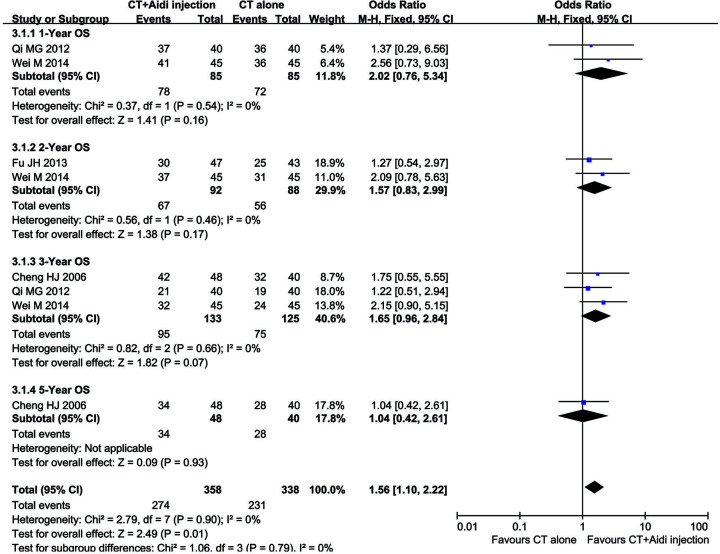
Forest plot of overall survival in patients treated with CT+Aidi injection and CT alone CT, Conventional treatment. The fixed effects meta-analysis model (Mantel–Haenszel method) was used.

Only four clinical trials [28,31,49,53] with 348 GT patients reported the OS (Figure 6). Although the meta-analysis revealed that the 1-year (OR = 2.02, 95% CI = 0.76–5.34, *P*=0.16), 2-year (OR = 1.57, 95% CI = 0.83–2.99, *P*=0.17), 3-year (OR = 1.65, 95% CI = 0.96–2.84, *P*=0.07), and 5-year (OR = 1.04, 95% CI = 0.42–2.61, *P*=0.93), OS rates of patients in the combined treatment group were greater than those of the control group, there were no statistically significant differences. Fixed-effect models were used to analyze the ORR due to low heterogeneity.

### QoL assessment

**Figure 7:**
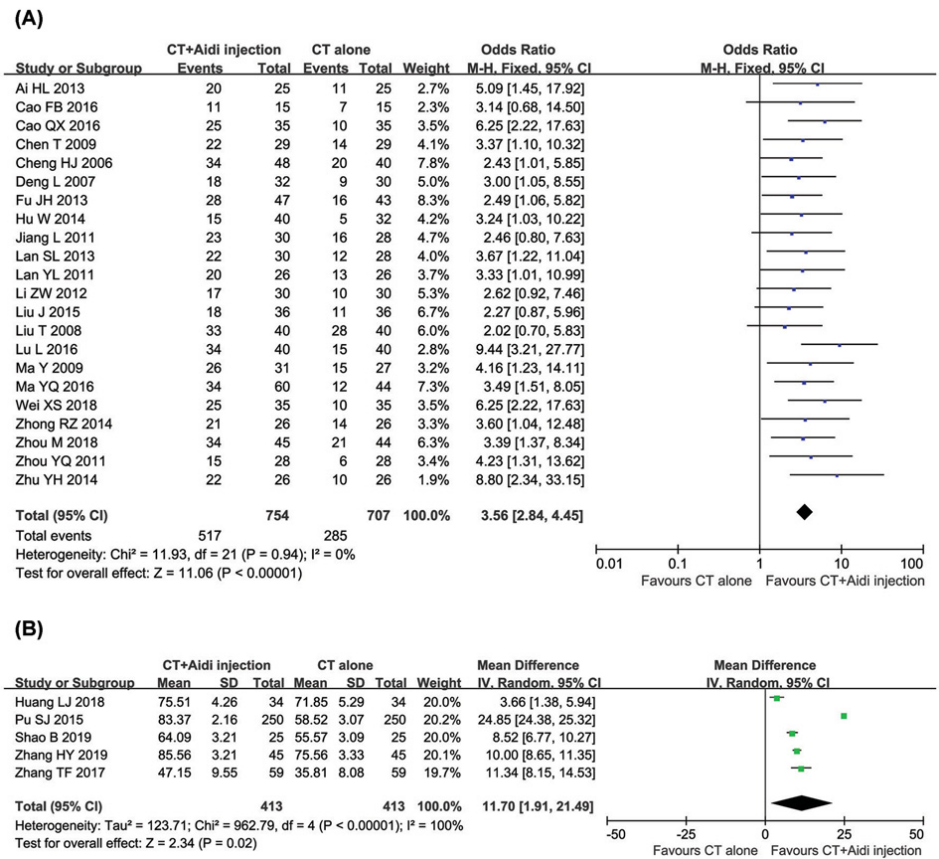
Forest plot of QIR and KPS in patients treated with CT+Aidi injection and CT alone (**A**) Forest plot of QIR; (**B**) Forest plot of KPS. CT, conventional treatment; KPS, Karnofsky score; QIR, Quality of life improved rate.

Twenty-two trials [24–28,30–32,35,37,38,40–43,45,46,54,58–61] with 1461 participants evaluated the QIR, and five trials [34,48,50,56,57] including 826 patients reported the KPS data (Figure 7). The results demonstrated that the QoL of GT patients in the combined group was significantly better than that in the control group, indicated by significantly increased QIR (OR = 3.56, 95% CI = 2.84–4.45, *P*<0.00001) and KPS (OR = 11.70, 95% CI = 1.91–21.49, *P*=0.02). Since the QIR (*P*=0.94, *I^2^* = 0%) was not heterogeneous among the studies, a fixed-effect model was used to analyze the OR; otherwise, a random-effect model was used.

### Evaluation of patient immunity

**Figure 8:**
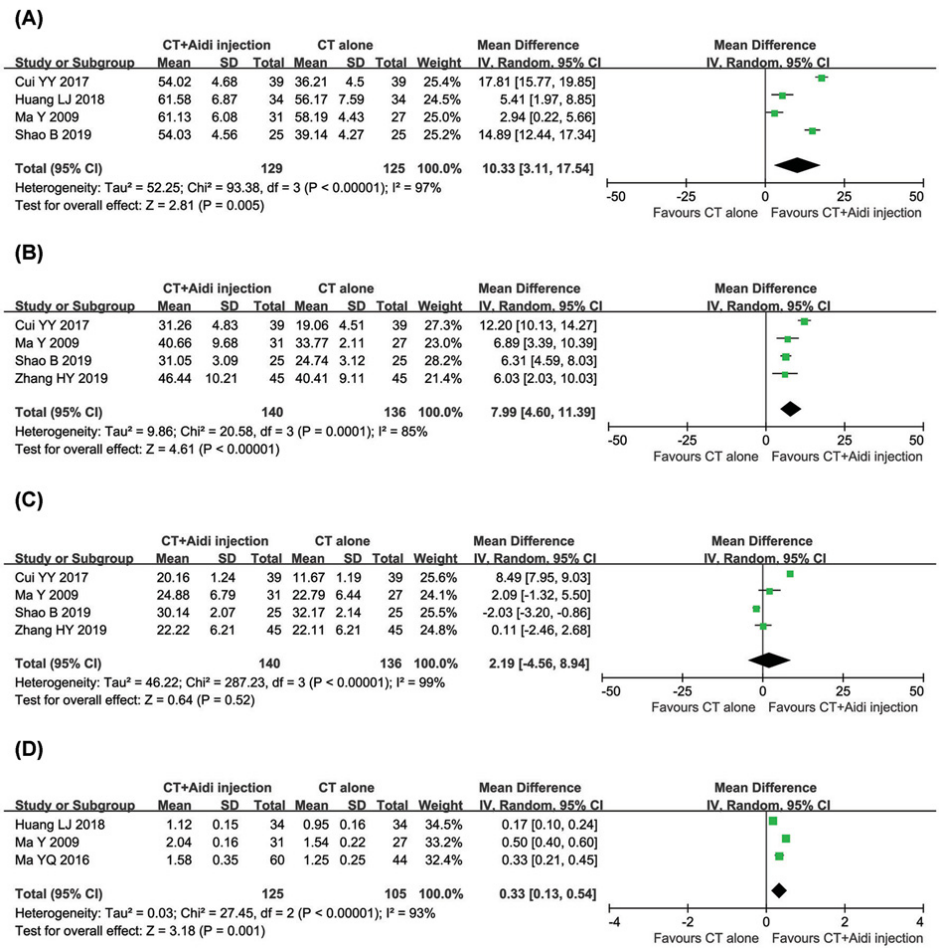
Forest plot of immune function in patients treated with CT+Aidi injection and CT alone (**A**) Forest plot of CD3^+^ percentage; (**B**) Forest plot of CD4^+^ percentage; (**C**) Forest plot of CD8^+^ percentage; (**D**) Forest plot of CD4^+^/CD8^+^ ratio; CT, Conventional treatment. The random effects meta-analysis model (inverse variance method) was used.

Differences in the immune status of patients between the two groups was examined in six controlled studies [29,34,45,46,50,56], which included a total of 448 patients (Figure 8). The percentages of CD3^+^ (OR = 10.33, 95% CI = 3.11–17.54, *P*=0.005) and CD4^+^ (OR = 7.99, 95% CI = 4.60–11.39, *P*<0.00001) and the CD4^+^/CD8^+^ ratios (OR = 0.33, 95% CI = 0.13–0.54, *P*=0.001) for the combined treatment group were significantly higher than those for the conventional treatment alone, whereas the CD8^+^ proportion (OR = 2.19, 95% CI = −4.56–8.94, *P*=0.52) did not significantly differ between the groups. A random effects model was used to pool this meta-analysis due to significant heterogeneity.

### Detection of tumor markers

**Figure 9:**
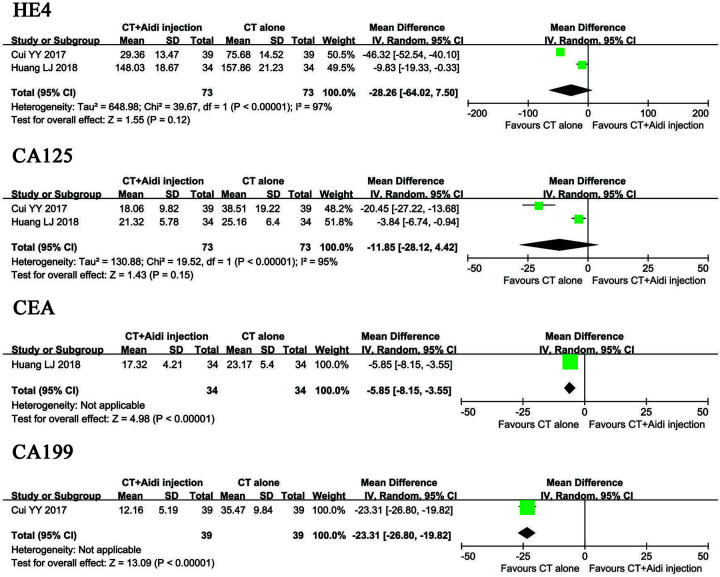
Forest plot of tumor markers in patients treated with CT+Aidi injection and CT alone HE4, Human epididymal protein 4; CA125, Cancer antigen 125; CEA, Carcinoembryonic antigen; CA199, Cancer antigen 199. CT, Conventional treatment. The random effects meta-analysis model (inverse variance method) was used.

Two clinical trials [29,34] with 448 patients evaluated tumor markers in GT patients for the two groups. As shown in Figure 9, HE4 levels (OR = -28.26, CI = -64.02–7.50, *P*=0.12), CA125 (OR = -11.85, CI = -28.12–4.42, *P*=0.15), CEA (OR = -5.85, CI = -8.15–3.55, *P*<0.00001), and CA199 (OR = -3.31, CI = -26.80–19.82, *P*<0.00001) decreased after combination therapy. However, there were no significant differences in HE4 and CA125 between groups. A random effects model was used to pool this meta-analysis due to significant heterogeneity.

### Assessment of adverse events

As shown in Supplementary Figure 1 and Table 3, patients treated with conventional methods combined with ADI exhibited lower incidences of gastrointestinal adverse effects (OR = 0.22, 95% CI = 0.16–0.31, *P*<0.00001), leukopenia (OR = 0.23, 95% CI = 0.16–0.32, *P*<0.00001), thrombocytopenia (OR = 0.31, 95% CI = 0.17–0.57, *P*=0.0001), hepatotoxicity (OR = 0.34, 95% CI = 0.23–0.50, *P*<0.00001), cardiotoxicity (OR = 0.23, 95% CI = 0.08–0.66, *P*=0.006), hematotoxicity (OR = 0.28, 95% CI = 0.16–0.51, *P*<0.0001), myelosuppression (OR = 0.47, 95% CI = 0.28–0.81, *P*=0.006), nausea and vomiting (OR = 0.50, 95% CI = 0.35–0.71, *P*=0.0001), and anemia (OR = 0.53, 95% CI = 0.30–0.94, *P*=0.03), whereas the incidence of nephrotoxicity (OR = 0.64, 95% CI = 0.27–1.47, *P*=0.29), diarrhea (OR = 0.59, 95% CI = 0.32–1.10, *P*=0.10), alopecia (OR = 0.68, 95% CI = 0.37–1.24, *P*=0.21), and neurotoxicity (OR = 0.52, 95% CI = 0.20–1.38, *P*=0.19) did not significantly differ between the groups. According to the heterogeneity test, statistical heterogeneity was observed for the incidence of thrombocytopenia (*P*=0.0008, *I^2^* = 65%) and neurotoxicity (*P*=0.09, *I^2^* = 58%), and a random effects model was used to pool this meta-analysis; otherwise, the fixed-effect model was used.

**Table 3:** Comparison of adverse events between the experimental and control group

Adverse events	Experimental group No. of patients (*n*)	Control group No. of patients (*n*)	Analysis method	Heterogeneity		Odds ratio (OR)	95% CI	*P*-value
				*I*^2^ (%)	*P*-value			
Gastrointestinal adverse effects	691	680	Fixed	0	0.77	0.22	0.16–0.31	<0.00001
Leukopenia	491	455	Fixed	30	0.15	0.23	0.16–0.32	<0.00001
Thrombocytopenia	443	423	Random	65	0.0008	0.31	0.17–0.57	0.0001
Hepatotoxicity	604	575	Fixed	0	0.46	0.34	0.23–0.50	<0.00001
Nephrotoxicity	107	87	Fixed	Not applicable		0.64	0.27–1.47	0.29
Cardiotoxicity	147	143	Fixed	0	0.93	0.23	0.08–0.66	0.006
Hematotoxicity	408	404	Fixed	0	0.88	0.28	0.16–0.51	<0.0001
Myelosuppression	320	276	Fixed	0	0.65	0.47	0.28–0.81	0.006
Nausea and vomiting	382	352	Fixed	50	0.05	0.50	0.35–0.71	0.0001
Anemia	131	111	Fixed	0	0.41	0.53	0.30–0.94	0.03
Diarrhea	108	89	Fixed	0	0.65	0.59	0.32–1.10	0.10
Alopecia	122	119	Fixed	39	0.19	0.68	0.37–1.24	0.21
Neurotoxicity	100	96	Random	58	0.09	0.52	0.20–1.38	0.19

**Notes:** Control group, Conventional treatments alone group; Experimental group, Conventional treatments and Aidi injection combined group.

### Publication bias

As shown in Supplementary Figure S2 and Table 4, funnel plots and the Begg’s and Egger’s regression tests showed that there was publication bias for the ORR (Begg = 0.032; Egger = 0.018) and thrombocytopenia incidence (Begg = 0.373; Egger = 0.031). To determine whether the bias affected the pooled risk of ORR and thrombocytopenia, a trim-and-fill analysis was performed. The adjusted OR indicated a trend similar to the results of the primary analysis (ORR, before: *P*<0.0001, after: *P*<0.0001; thrombocytopenia, before: *P*<0.0001, after: *P*<0.0001), indicating that the primary conclusions were reliable. Parameters reported in less than 10 papers were not used in the publication bias analysis.

**Table 4:** Publication bias on therapeutic efficacy and adverse events

Publication Bias	Therapeutic efficacy			Adverse events			
	ORR	DCR	QIR	Gastrointestinal adverse effects	Leukopenia	Thrombocytopenia	Hepatotoxicity
**Begg**	0.032	0.747	0.150	0.274	1.000	0.373	0.152
**Egger**	0.018	0.947	0.116	0.231	0.149	0.031	0.314
**Trim and fill analysis**							
**before**	*P*<0.0001					*P*<0.0001	
**after**	*P*<0.0001					*P*<0.0001	

**Notes:** Parameters discussed in over 10 papers were conducted bias analyses.

Abbreviations: DCR, disease control rate; ORR, overall response rate; QIR, quality of life improved rate.

### Sensitivity analysis

As shown in Supplementary Figure S3, the results revealed that no individual studies significantly affected the primary outcomes, indicating statistically robust results. Parameters reported in less than 10 studies were not included in the sensitivity analysis.

We also conducted subgroup analyses for tumor type, the dosage of ADI, sample size, and study type. As shown in Table 5, our analysis revealed no significant differences in dosage of ADI, sample size, and study type. Further, combination therapy was more likely to improve the QoL of patients with OC and CC, as compared with EC patients. However, since only one study [35] reported the effect of combination therapy on the QoL of EC patients, these results cannot be generalized.

**Table 5:** Subgroup analyses of ORR, DCR and QIR between the experimental and control group

Parameter	Factors at study level	Experimental group No. of patients (*n*)	Control group No. of patients (*n*)	Analysis method	Heterogeneity		Risk ratio (RR)	95% CI	*P*-value
					*I*^2^ (%)	*P*-value			
**ORR**	**Tumor types**								
	OC	885	810	Fixed	0	0.91	2.34	1.89–2.90	<0.00001
	CC	280	225	Random	51	0.08	2.55	1.35–4.80	0.004
	EC	34	34	Fixed	Not applicable		2.96	1.10–7.94	0.03
	**Study types**								
	RCT	963	900	Fixed	0	0.95	2.58	2.09–3.19	<0.00001
	Non-RCT	283	214	Fixed	40	0.15	1.91	1.32–2.78	0.0006
	**Dosage of Aidi injection**								
	=50 ml/day	506	496	Fixed	0	0.98	2.66	2.01–3.52	<0.00001
	>50 ml/day	469	447	Fixed	0	0.48	2.30	1.70–3.10	<0.00001
	**Study sample size**								
	≥60	960	835	Fixed	0	0.58	2.28	1.85–2.82	<0.00001
	<60	286	279	Fixed	0	0.87	2.80	1.93–4.05	<0.00001
**DCR**	**Tumor types**								
	OC	637	618	Fixed	0	0.93	2.69	1.93–3.75	<0.00001
	CC	221	166	Fixed	0	0.52	1.86	1.11–3.13	0.02
	EC	34	34	Fixed	Not applicable		4.31	1.07–17.39	0.04
	**Study types**								
	RCT	740	680	Fixed	0	0.93	2.77	2.02–3.81	<0.00001
	Non-RCT	184	168	Fixed	0	0.44	2.13	1.31–3.45	0.002
	**Dosage of Aidi injection**								
	50 ml/day	381	374	Fixed	0	0.99	2.86	1.81–4.53	<0.00001
	>50 ml/day	430	408	Fixed	6	0.39	2.58	1.82–3.67	<0.00001
	**Study sample size**								
	≥60	653	584	Random	0	0.75	2.57	1.91–3.45	<0.00001
	<60	271	264	Random	0	0.85	2.57	1.42–4.63	0.002
**QIR**	**Tumor types**								
	OC	531	512	Fixed	0	0.81	3.71	2.84–4.85	<0.00001
	CC	136	112	Fixed	0	0.73	3.18	1.86–5.43	<0.0001
	EC	30	28	Fixed	Not applicable		2.46	0.80–7.63	0.12
	**Study types**								
	RCT	612	585	Fixed	0	0.93	3.56	2.77–4.57	<0.00001
	Non-RCT	142	122	Fixed	0	0.40	3.56	2.13–5.95	<0.00001
	**Dosage of Aidi injection**								
	50 ml/day	272	261	Fixed	0	1.00	2.90	2.02–4.17	<0.00001
	>50 ml/day	427	399	Fixed	0	0.62	4.14	3.06–5.61	<0.00001
	**Study sample size**								
	≥60	488	449	Fixed	0	0.61	3.39	2.57–4.48	<0.00001
	<60	266	258	Fixed	0	0.98	3.89	2.66–5.70	<0.00001

**Notes:** Control group: conventional treatments alone group; Experimental group: conventional treatments and Aidi injection combined group.

**Abbreviations:** DCR: disease control rate; ORR: overall response rate; QIR: quality of life improved rate.

## Discussion

As a type of traditional Chinese biomedical preparation, ADI has been clinically applied as an effective adjuvant drug in cancer treatment for decades [18–20]. Although several studies have reported that addition of ADI could be beneficial to patients with GT [24–61], the exact therapeutic effects have yet to be systematically evaluated. Thus, in-depth knowledge of the efficacy and safety of ADI is needed. This systematic review provides evidence that clinicians can reference for the development of the most effective postoperative adjuvant treatment strategy for patients with OC, CC, or EC. These results may also provide the foundation for further research in this area.

Data from 38 trials [24–61] including a total of 3309 GT patients were used in our meta-analysis. The pooled results revealed that ADI in combination with conventional GT treatment was more beneficial than conventional treatment alone. Moreover, ADI significantly improved the ORR, DCR, and QoL in GT patients (*P*<0.05) compared with conventional treatment alone. Among the included studies, four also assessed whether ADI could increase the long-term survival rates in GT patients. Although the results showed that the 1- 2-, 3-, and 5-year OS rates of patients in the combined treatment group were greater than those of the control group, significant differences were not observed. Specific molecular markers including HE4, CA125, CEA, and CA199 are commonly used to predict the recurrence, metastasis, and prognosis of GT after treatment [71,72]. Our analysis showed that these tumor markers decreased after combination treatment, but HE4 and CA125 levels did not significantly differ between groups. Overall, these results indicated that ADI could improve the curative effects of conventional treatment methods to some extent.

T lymphocyte subsets (CD3^+^, CD4^+^, and CD8^+^ cell subsets) and CD4^+^/CD8^+^ ratio play an important role in antitumor immunity [22,23]. Several studies have reported that ADI can enhance the body’s immunity and resistance to tumors [22,23]. Our analysis demonstrated that the percentages of CD3^+^ and CD4^+^ and the CD4^+^/CD8^+^ ratios were all significantly increased in GT patients treated with ADI, indicating that immune function of GT patients improved after ADI adjuvant therapy.

Safety is the top priority in clinical treatment. Twenty-six clinical trials [25–28,31–37,39,41–48,52,55,56,59–61] with a total of 2415 GT patients reported adverse events, as defined by the World Health Organization standards. Meta-analysis revealed that patients who received ADI in combination with conventional treatment demonstrated a lower risk for gastrointestinal adverse effects, leukopenia, thrombocytopenia, hepatotoxicity, cardiotoxicity, hematotoxicity, myelosuppression, nausea and vomiting, and anemia, as compared with those who underwent conventional treatment alone. However, the incidence of other toxic side effects did not significantly differ between groups. Therefore, ADI appears to be a safe auxiliary anti-tumor drug for GT patients, and it can alleviate some of the adverse events associated with conventional treatment.

There were a few limitations to our analysis. First, there was publication bias for some indicators, as authors tend to report favorable results to editors. Second, different trials evaluated the treatment efficacy using different outcomes, resulting in a reduced sample size, which made it difficult to summarize results across studies using the same scale. Third, allocation concealment and blinding method were not clear in most of the included studies, which could have resulted in exaggerated estimates of the treatment effect. Finally, most of the included trials assessed efficacy immediately after the completion of treatment. Therefore, methodologically rigorous trials are needed to assess the long-term effects of ADI on OC, CC, and EC. Given these limitations, some of the findings of our study should be cautiously interpreted.

## Conclusion

In summary, findings of this meta-analysis indicate that ADI in combination with conventional treatment is effective in treating patients with OC, CC, and EC. The clinical application of ADI in such patients not only clearly enhanced the therapeutic effects of conventional treatment, but also effectively improved the QoL and immune function. Thus, we anticipate that our study will provide valuable evidence for further evaluation of ADI. On the other hand, considering that only a few clinical trials evaluated the long-term efficacy and immune-regulatory effect of ADI, additional studies with high-quality evidence are needed to verify the effectiveness of ADI-mediated therapy for GT.

## Supplementary Material

Supplementary Figures S1-S3

## Data Availability

All supporting data are included within the main article and its supplementary files.

## Abbreviations

ADI: Aidi injection
CA125: Cancer antigen 125
CA199: Cancer antigen 199
CBM: Chinese Biological Medicine Database
CC: cervical cancer
CEA: carcinoembryonic antigen
CI: confidence interval
CNKI: China National Knowledge Infrastructure
CSJD: Chinese Scientific Journal Database
DCR: disease control rate
EC: endometrial cancer
GT: gynecologic tumor
HE4: Human epididymal protein 4
KPS: Karnofsky Performance Score
MINRRS: Methodological Index for Non-randomized Studies
OC: ovarian cancer
OR: odds ratio
ORR: overall response rate
OS: overall survival
PRISMA: Preferred Reporting Items for Systematic Reviews and Meta-Analyses
QIR: quality of life improved rate
QoL: quality of life
RECIST: Response Evaluation Criteria in Solid Tumors
SFDA: State Food and Drug Administration
