# Supplementary material for: Aidi injection, a traditional Chinese biomedical preparation for gynecologic tumors: a systematic review and PRISMA-compliant meta-analysis
Source: Biosci Rep. 2021 Mar 5;41(3):BSR20204457. doi: 10.1042/BSR20204457 (PMC7937908; doi:10.1042/BSR20204457)
Supplement: Supplementary Figures S1-S3 [file BSR-2020-4457_supp.pdf]

A

Study or Subgroup	CT alone Events	CT alone Total	CT+Aidi injection Events	CT+Aidi injection Total	Weight	Odds Ratio M-H, Fixed, 95% CI
Cao FB 2016	2	15	5	15	2.7%	0.31 [0.05, 1.93]
Cao QX 2016	9	35	25	35	11.7%	0.14 [0.05, 0.40]
Chen T 2009	18	29	25	29	6.0%	0.26 [0.07, 0.96]
Fu JH 2013	1	47	6	43	3.9%	0.13 [0.02, 1.16]
Huang LJ 2018	7	34	15	34	7.5%	0.33 [0.11, 0.96]
Jiang L 2011	22	30	24	28	4.2%	0.46 [0.12, 1.74]
Liu J 2015	3	36	6	36	3.5%	0.45 [0.10, 1.98]
Lu L 2016	2	40	6	40	3.6%	0.30 [0.06, 1.58]
Ma Y 2009	26	31	26	27	2.8%	0.20 [0.02, 1.83]
Pu SJ 2015	11	250	39	250	23.5%	0.25 [0.12, 0.50]
Zhang HY 2019	2	45	14	45	8.4%	0.10 [0.02, 0.49]
Zhou M 2018	28	45	38	44	9.1%	0.26 [0.09, 0.74]
Zhou YQ 2011	11	28	28	28	10.8%	0.01 [0.00, 0.21]
Zhu YH 2014	1	26	4	26	2.4%	0.22 [0.02, 2.12]
**Total (95% CI)**		**691**		**680**	**100.0%**	**0.22 [0.16, 0.31]**
Total events	143		261			

Heterogeneity: Chi² = 9.07, df = 13 (P = 0.77); I² = 0%

Test for overall effect: Z = 8.74 (P < 0.00001)

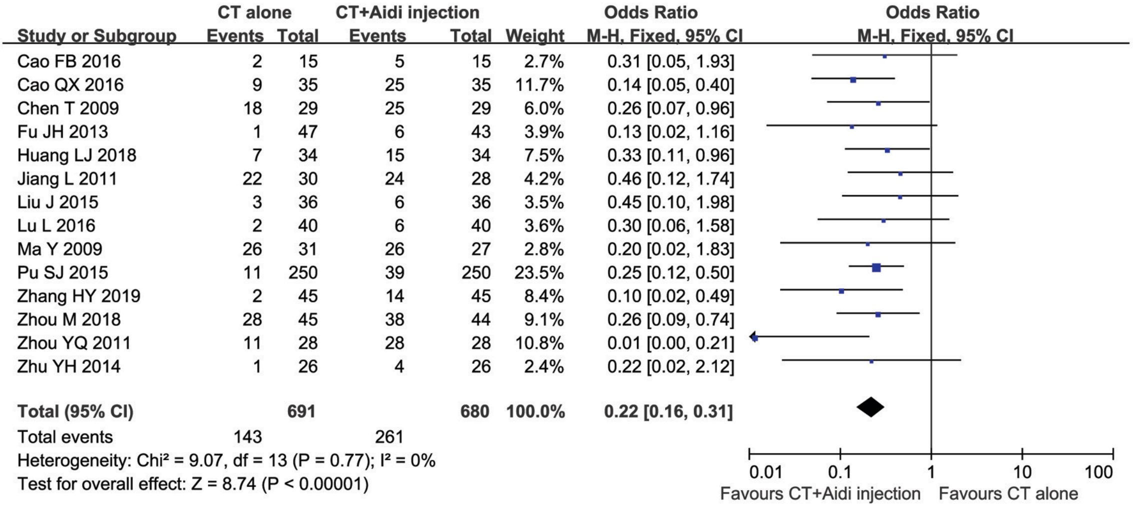

B

Study or Subgroup	CT alone Events	CT alone Total	CT+Aidi injection Events	CT+Aidi injection Total	Weight	Odds Ratio M-H, Fixed, 95% CI
Cao FB 2016	2	15	5	15	3.0%	0.31 [0.05, 1.93]
Cao QX 2016	9	35	21	35	10.7%	0.23 [0.08, 0.64]
Chen T 2009	17	29	25	29	7.1%	0.23 [0.06, 0.82]
Cheng HJ 2006	28	48	35	40	10.9%	0.20 [0.07, 0.60]
Hu W 2014	6	40	4	32	2.6%	1.24 [0.32, 4.82]
Lan GH 2017	3	29	10	29	6.1%	0.22 [0.05, 0.91]
Lan SL 2013	9	30	18	28	8.9%	0.24 [0.08, 0.71]
Li YF 2007	3	21	16	20	9.6%	0.04 [0.01, 0.22]
Liu J 2015	2	36	7	36	4.5%	0.24 [0.05, 1.27]
Ma YQ 2016	44	60	40	44	8.4%	0.28 [0.08, 0.89]
Nian L 2019	4	75	7	75	4.5%	0.55 [0.15, 1.95]
Zhou M 2018	27	45	38	44	10.5%	0.24 [0.08, 0.68]
Zhou YQ 2011	9	28	28	28	13.1%	0.01 [0.00, 0.16]
**Total (95% CI)**		**491**		**455**	**100.0%**	**0.23 [0.16, 0.32]**
Total events	163		254			

Heterogeneity: Chi² = 17.07, df = 12 (P = 0.15); I² = 30%

Test for overall effect: Z = 8.45 (P < 0.00001)

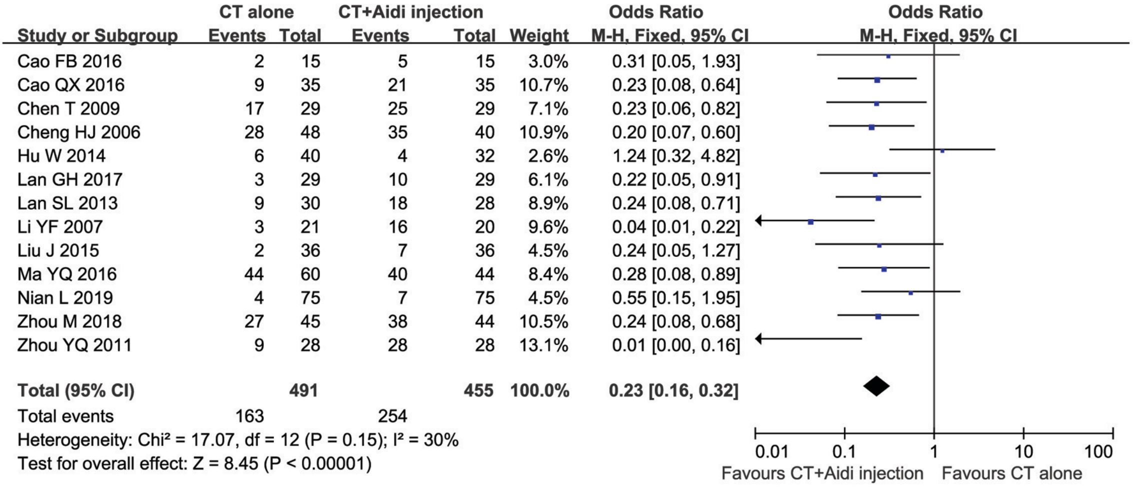

C

Study or Subgroup	CT alone Events	CT alone Total	CT+Aidi injection Events	CT+Aidi injection Total	Weight	Odds Ratio M-H, Random, 95% CI
Cao FB 2016	2	15	5	15	5.9%	0.31 [0.05, 1.93]
Cao QX 2016	5	35	20	35	8.9%	0.13 [0.04, 0.40]
Chen T 2009	19	29	24	29	8.5%	0.40 [0.12, 1.36]
Lan GH 2017	4	29	11	29	8.2%	0.26 [0.07, 0.96]
Lan SL 2013	11	30	20	28	9.1%	0.23 [0.08, 0.70]
Li YF 2007	4	21	14	20	7.5%	0.10 [0.02, 0.43]
Liu J 2015	3	36	7	36	7.5%	0.38 [0.09, 1.59]
Liu T 2008	5	40	8	40	8.6%	0.57 [0.17, 1.93]
Ma YQ 2016	38	60	22	44	10.8%	1.73 [0.78, 3.81]
Nian L 2019	3	75	5	75	7.4%	0.58 [0.13, 2.53]
Zhou M 2018	30	45	36	44	9.8%	0.44 [0.17, 1.19]
Zhou YQ 2011	4	28	22	28	7.8%	0.05 [0.01, 0.18]
**Total (95% CI)**		**443**		**423**	**100.0%**	**0.31 [0.17, 0.57]**
Total events	128		194			

Heterogeneity: Tau² = 0.72; Chi² = 31.79, df = 11 (P = 0.0008); I² = 65%

Test for overall effect: Z = 3.80 (P = 0.0001)

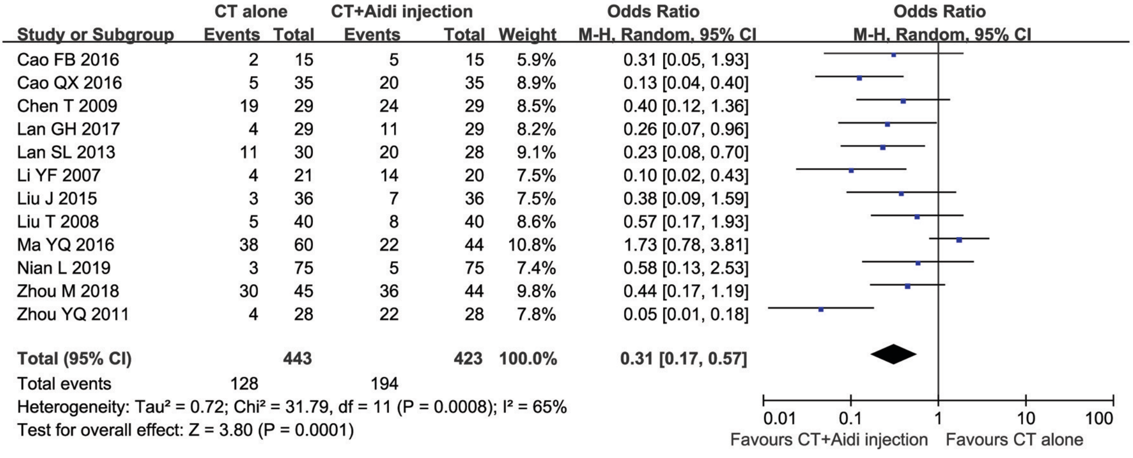

D

Study or Subgroup	CT alone Events	CT alone Total	CT+Aidi injection Events	CT+Aidi injection Total	Weight	Odds Ratio M-H, Fixed, 95% CI
Fu JH 2013	1	47	1	43	1.0%	0.91 [0.06, 15.06]
Lan SL 2013	7	30	16	28	13.0%	0.23 [0.07, 0.71]
Liu T 2008	3	40	5	40	4.7%	0.57 [0.13, 2.55]
Lu L 2016	2	40	3	40	2.9%	0.65 [0.10, 4.11]
Lv J 2003	5	35	15	32	13.7%	0.19 [0.06, 0.61]
Ma Y 2009	5	31	11	27	10.1%	0.28 [0.08, 0.95]
Ma YQ 2016	16	60	14	44	12.1%	0.78 [0.33, 1.83]
Pu SJ 2015	9	250	38	250	37.5%	0.21 [0.10, 0.44]
Zhang HY 2019	2	45	3	45	2.9%	0.65 [0.10, 4.10]
Zhu YH 2014	1	26	2	26	2.0%	0.48 [0.04, 5.65]
**Total (95% CI)**		**604**		**575**	**100.0%**	**0.34 [0.23, 0.50]**
Total events	51		108			

Heterogeneity: Chi² = 8.75, df = 9 (P = 0.46); I² = 0%
Test for overall effect: Z = 5.60 (P < 0.00001)

Odds Ratio M-H, Fixed, 95% CI

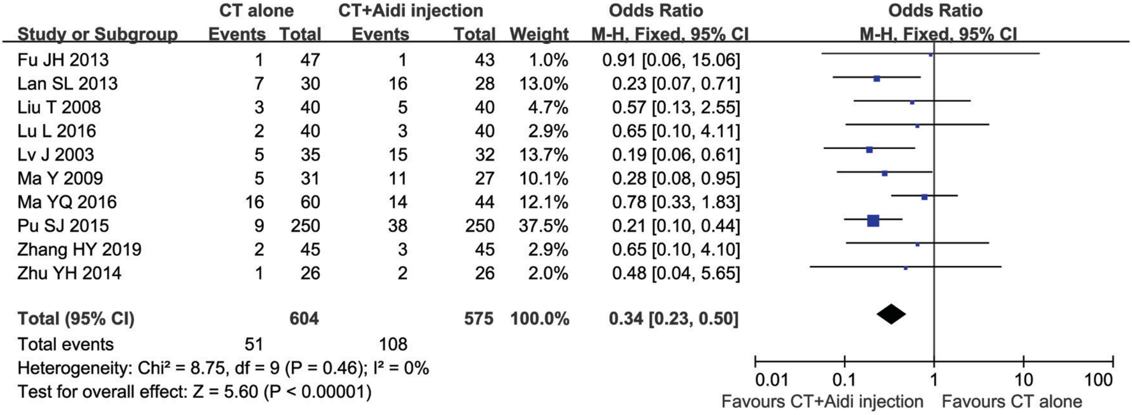

E

Study or Subgroup	CT alone Events	CT alone Total	CT+Aidi injection Events	CT+Aidi injection Total	Weight	Odds Ratio M-H, Fixed, 95% CI
Fu JH 2013	0	47	0	43		Not estimable
Ma YQ 2016	16	60	16	44	100.0%	0.64 [0.27, 1.47]
**Total (95% CI)**		**107**		**87**	**100.0%**	**0.64 [0.27, 1.47]**
Total events	16		16			

Heterogeneity: Not applicable
Test for overall effect: Z = 1.06 (P = 0.29)

Odds Ratio M-H, Fixed, 95% CI

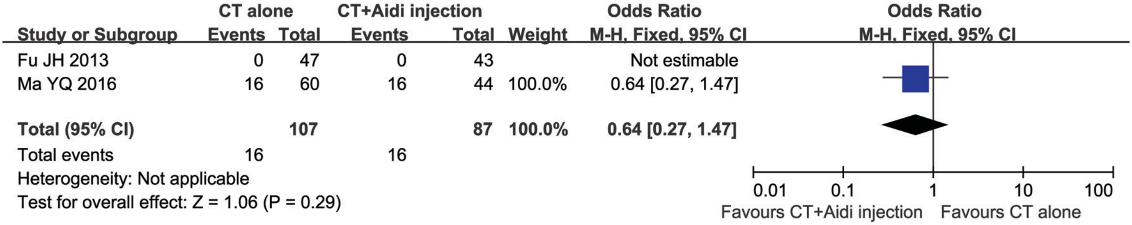

F

Study or Subgroup	CT alone Events	CT alone Total	CT+Aidi injection Events	CT+Aidi injection Total	Weight	Odds Ratio M-H, Fixed, 95% CI
Fu JH 2013	1	47	2	43	12.0%	0.45 [0.04, 5.10]
Huang LJ 2018	3	34	10	34	53.4%	0.23 [0.06, 0.94]
Lu L 2016	0	40	3	40	20.3%	0.13 [0.01, 2.65]
Zhu YH 2014	0	26	2	26	14.4%	0.18 [0.01, 4.05]
**Total (95% CI)**		**147**		**143**	**100.0%**	**0.23 [0.08, 0.66]**
Total events	4		17			

Heterogeneity: Chi² = 0.43, df = 3 (P = 0.93); I² = 0%
Test for overall effect: Z = 2.75 (P = 0.006)

Odds Ratio M-H, Fixed, 95% CI

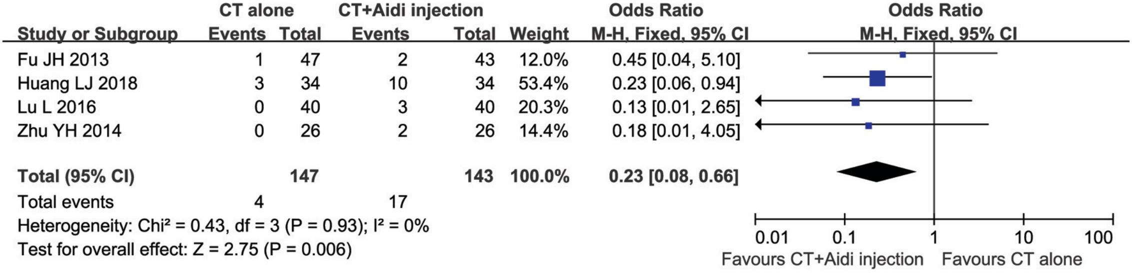

G

Study or Subgroup	CT alone Events	CT alone Total	CT+Aidi injection Events	CT+Aidi injection Total	Weight	Odds Ratio M-H, Fixed, 95% CI
Fu JH 2013	2	47	8	43	17.2%	0.19 [0.04, 0.97]
Lu L 2016	1	40	5	40	10.5%	0.18 [0.02, 1.61]
Pu SJ 2015	9	250	29	250	60.0%	0.28 [0.13, 0.61]
Zhang HY 2019	2	45	3	45	6.2%	0.65 [0.10, 4.10]
Zhu YH 2014	1	26	3	26	6.2%	0.31 [0.03, 3.16]
**Total (95% CI)**		**408**		**404**	**100.0%**	**0.28 [0.16, 0.51]**
Total events	15		48			

Heterogeneity: Chi² = 1.17, df = 4 (P = 0.88); I² = 0%
Test for overall effect: Z = 4.16 (P < 0.0001)

Odds Ratio M-H, Fixed, 95% CI

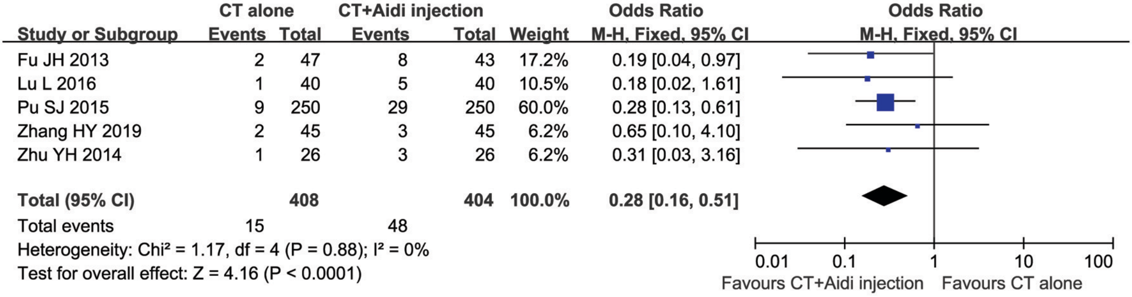

H

Study or Subgroup	CT alone Events	CT alone Total	CT+Aidi injection Events	CT+Aidi injection Total	Weight	Odds Ratio M-H, Fixed, 95% CI
Hu YF 2011	63	73	32	34	14.4%	0.39 [0.08, 1.91]
Huang LJ 2018	4	34	11	34	23.4%	0.28 [0.08, 0.99]
Jiang L 2011	27	30	28	28	8.0%	0.14 [0.01, 2.79]
Nian L 2019	2	75	6	75	14.1%	0.32 [0.06, 1.61]
Wang YF 2006	21	48	22	45	30.8%	0.81 [0.36, 1.84]
Yu J 2015	2	60	4	60	9.3%	0.48 [0.09, 2.74]
**Total (95% CI)**		**320**		**276**	**100.0%**	**0.47 [0.28, 0.81]**
Total events	119		103			

Heterogeneity: Chi² = 3.30, df = 5 (P = 0.65); I² = 0%
Test for overall effect: Z = 2.76 (P = 0.006)

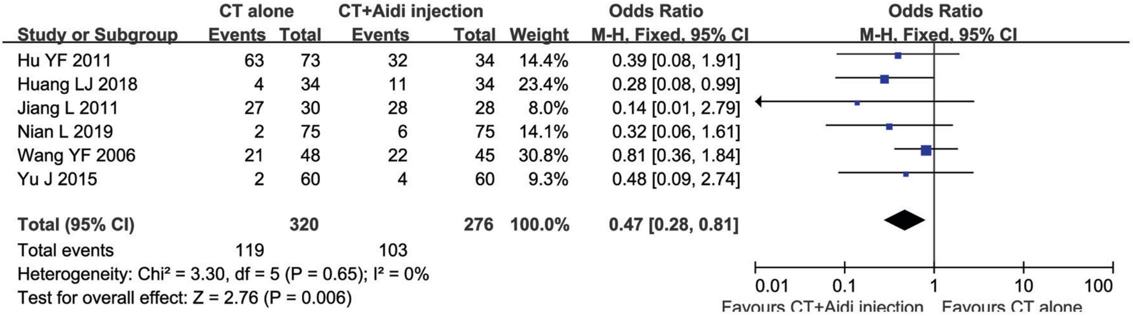

I

Study or Subgroup	CT alone Events	CT alone Total	CT+Aidi injection Events	CT+Aidi injection Total	Weight	Odds Ratio M-H, Fixed, 95% CI
Cheng HJ 2006	19	48	29	40	22.1%	0.25 [0.10, 0.61]
Lan SL 2013	14	30	13	28	8.3%	1.01 [0.36, 2.84]
Li YF 2007	3	21	14	20	14.2%	0.07 [0.02, 0.34]
Liu T 2008	18	40	19	40	12.1%	0.90 [0.38, 2.18]
Ma YQ 2016	24	60	28	44	22.4%	0.38 [0.17, 0.85]
Nian L 2019	3	75	6	75	6.6%	0.48 [0.12, 1.99]
Wang YF 2006	13	48	14	45	12.2%	0.82 [0.34, 2.02]
Yu J 2015	2	60	2	60	2.2%	1.00 [0.14, 7.34]
**Total (95% CI)**		**382**		**352**	**100.0%**	**0.50 [0.35, 0.71]**
Total events	96		125			

Heterogeneity: Chi² = 13.96, df = 7 (P = 0.05); I² = 50%
Test for overall effect: Z = 3.85 (P = 0.0001)

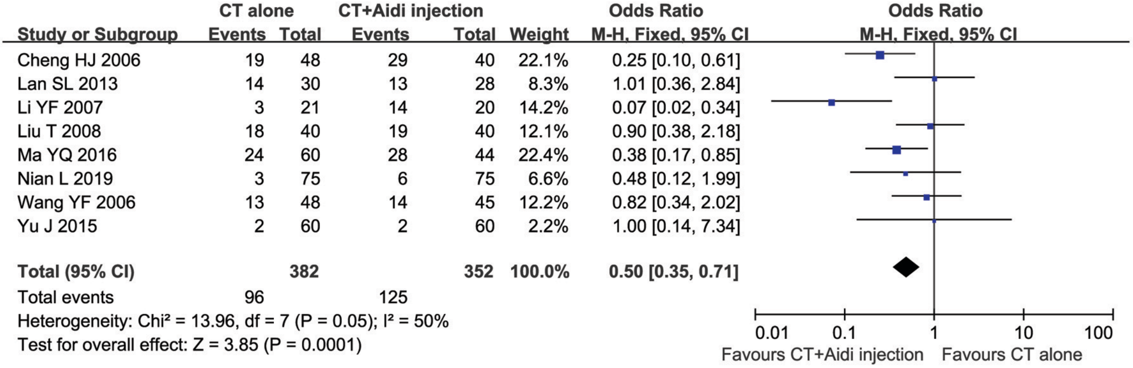

J

Study or Subgroup	CT alone Events	CT alone Total	CT+Aidi injection Events	CT+Aidi injection Total	Weight	Odds Ratio M-H, Fixed, 95% CI
Liu T 2008	9	40	11	40	26.1%	0.77 [0.28, 2.11]
Ma Y 2009	4	31	10	27	28.6%	0.25 [0.07, 0.93]
Ma YQ 2016	36	60	32	44	45.3%	0.56 [0.24, 1.30]
**Total (95% CI)**		**131**		**111**	**100.0%**	**0.53 [0.30, 0.94]**
Total events	49		53			

Heterogeneity: Chi² = 1.76, df = 2 (P = 0.41); I² = 0%
Test for overall effect: Z = 2.19 (P = 0.03)

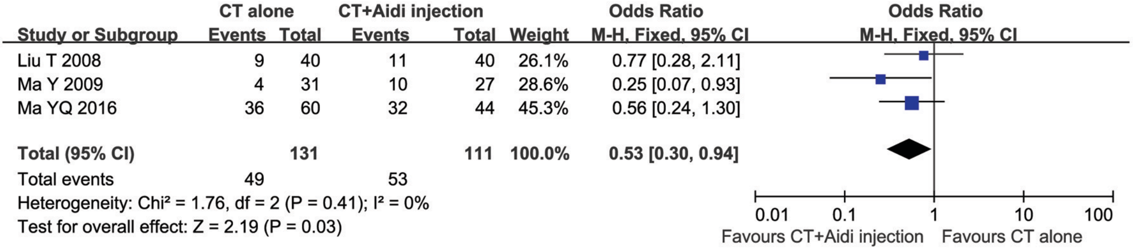

K

Study or Subgroup	CT alone Events	CT alone Total	CT+Aidi injection Events	CT+Aidi injection Total	Weight	Odds Ratio M-H, Fixed, 95% CI
Ma YQ 2016	26	60	26	44	64.8%	0.53 [0.24, 1.16]
Wang YF 2006	9	48	11	45	35.2%	0.71 [0.26, 1.93]
**Total (95% CI)**		**108**		**89**	**100.0%**	**0.59 [0.32, 1.10]**
Total events	35		37			

Heterogeneity: Chi² = 0.21, df = 1 (P = 0.65); I² = 0%
Test for overall effect: Z = 1.65 (P = 0.10)

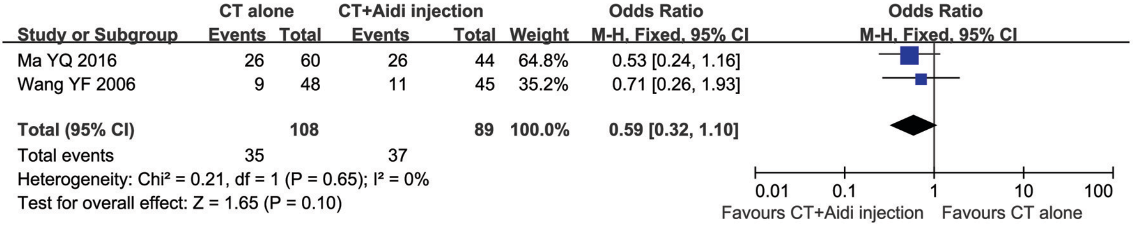

L

Study or Subgroup	CT alone Events	CT alone Total	CT+Aidi injection Events	CT+Aidi injection Total	Weight	Odds Ratio M-H, Fixed, 95% CI
Huang LJ 2018	5	34	13	34	42.6%	0.28 [0.09, 0.90]
Liu T 2008	21	40	22	40	40.1%	0.90 [0.38, 2.18]
Wang YF 2006	6	48	5	45	17.3%	1.14 [0.32, 4.04]
**Total (95% CI)**		**122**		**119**	**100.0%**	**0.68 [0.37, 1.24]**
Total events	32		40			

Heterogeneity: $Chi^2 = 3.27$, df = 2 (P = 0.19); $I^2 = 39\%$
Test for overall effect: Z = 1.27 (P = 0.21)

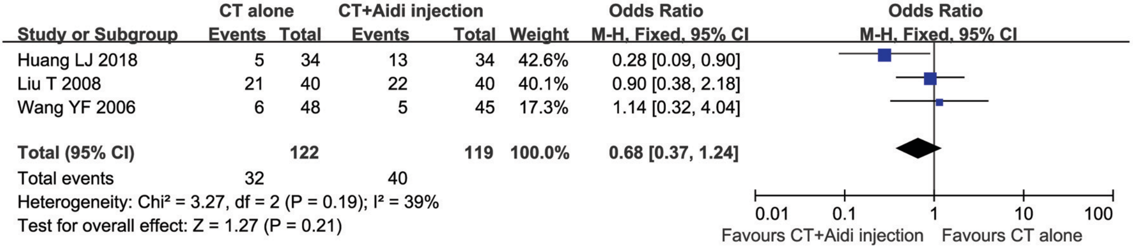

M

Study or Subgroup	CT alone Events	CT alone Total	CT+Aidi injection Events	CT+Aidi injection Total	Weight	Odds Ratio M-H, Random, 95% CI
Jiang L 2011	21	30	23	28	29.6%	0.51 [0.15, 1.76]
Lan SL 2013	12	30	21	28	32.4%	0.22 [0.07, 0.68]
Liu T 2008	15	40	14	40	38.0%	1.11 [0.45, 2.77]
**Total (95% CI)**		**100**		**96**	**100.0%**	**0.52 [0.20, 1.38]**
Total events	48		58			

Heterogeneity: $Tau^2 = 0.43$; $Chi^2 = 4.80$, df = 2 (P = 0.09); $I^2 = 58\%$
Test for overall effect: Z = 1.30 (P = 0.19)

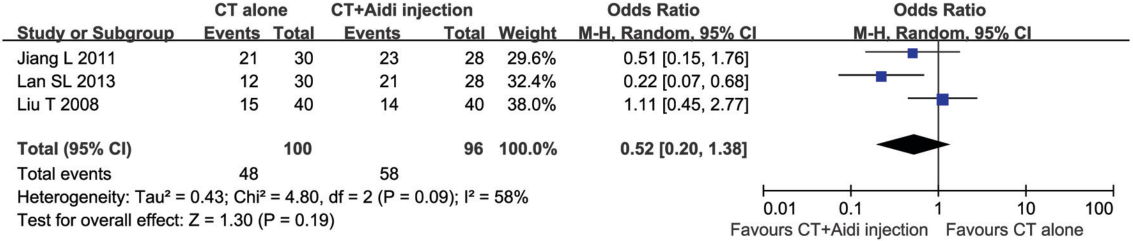

Supplement Figure 1. Forest plot of adverse effects in patients treated with CT+Aidi injection and CT alone.

(A) Forest plot of gastrointestinal adverse effects; (B) Forest plot of leukopenia; (C) Forest plot of thrombocytopenia; (D) Forest plot of hepatotoxicity; (E) Forest plot of nephrotoxicity; (F) Forest plot of cardiotoxicity; (G) Forest plot of hematotoxicity; (H) Forest plot of myelosuppression; (I) Forest plot of nausea and vomiting; (J) Forest plot of anemia; (K) Forest plot of diarrhea; (L) Forest plot of alopecia; (M) Forest plot of neurotoxicity. CT, Conventional treatment.

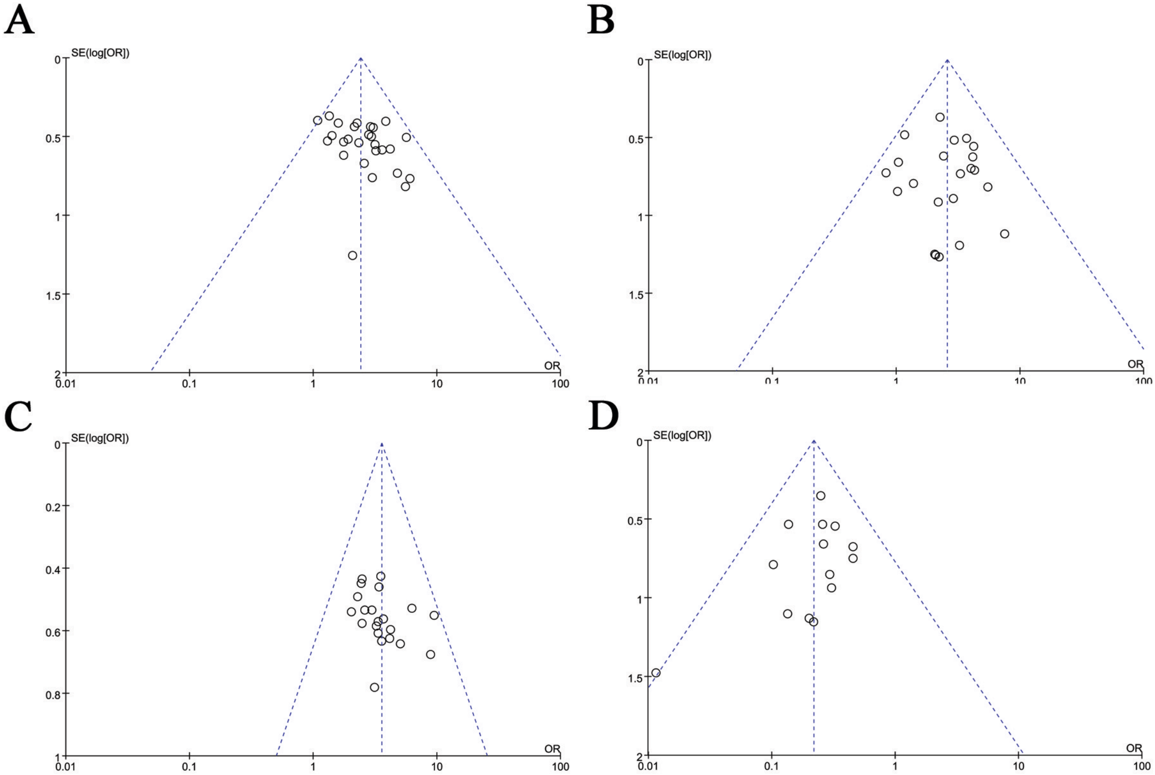

A
SE(log[OR])
0
0.5
1
1.5
2
0.01
0.1
1
10
100
OR
B
SE(log[OR])
0
0.5
1
1.5
2
0.01
0.1
1
10
100
OR
C
SE(log[OR])
0
0.2
0.4
0.6
0.8
1
0.01
0.1
1
10
100
OR
D
SE(log[OR])
0
0.5
1
1.5
2
0.01
0.1
1
10
100
OR

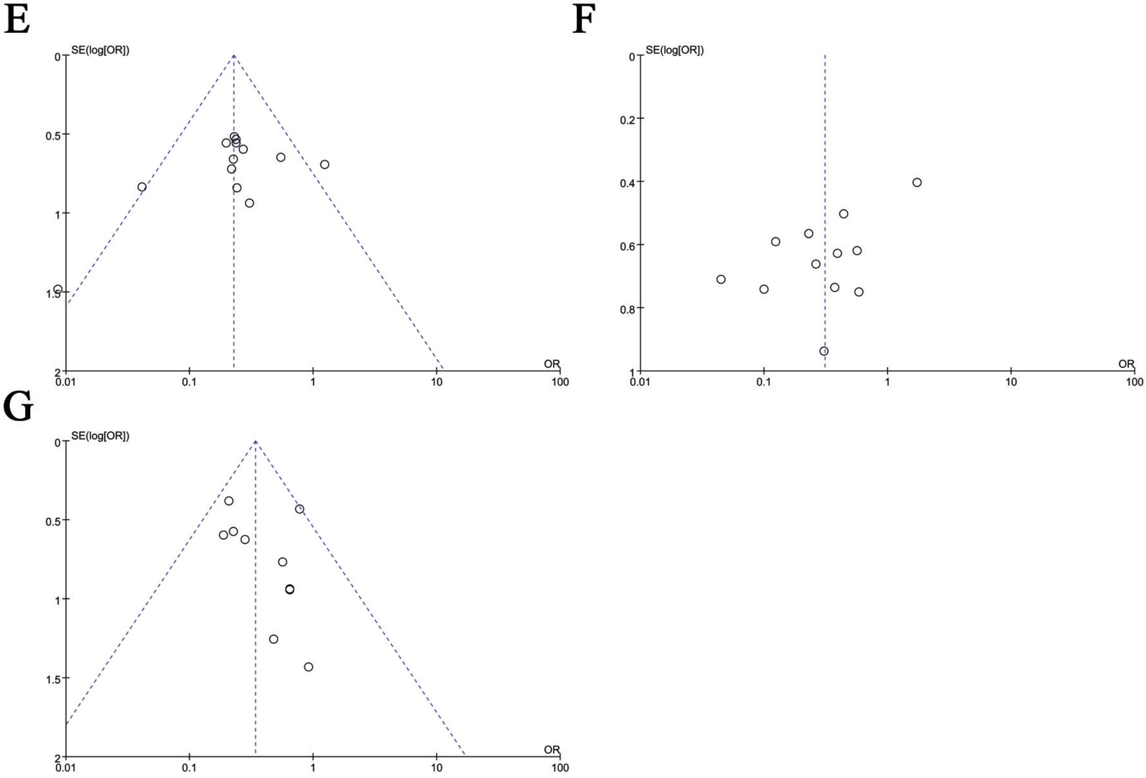

E
SE(log[OR])
0
0.5
1
1.5
2
OR
0.01
0.1
1
10
100
F
SE(log[OR])
0
0.2
0.4
0.6
0.8
1
OR
0.01
0.1
1
10
100
G
SE(log[OR])
0
0.5
1
1.5
2
OR
0.01
0.1
1
10
100

Supplement Figure 2. Funnel plot for the publication bias

(A) Funnel plot of overall response rate; (B) Funnel plot of disease control rate; (C) Funnel plot of quality of life improved rate; (D) Funnel plot of gastrointestinal adverse effects; (E) Funnel plot of leukopenia; (F) Funnel plot of thrombocytopenia; (G) Funnel plot of hepatotoxicity. Parameters discussed less than 10 papers were not conducted publication bias analyses.

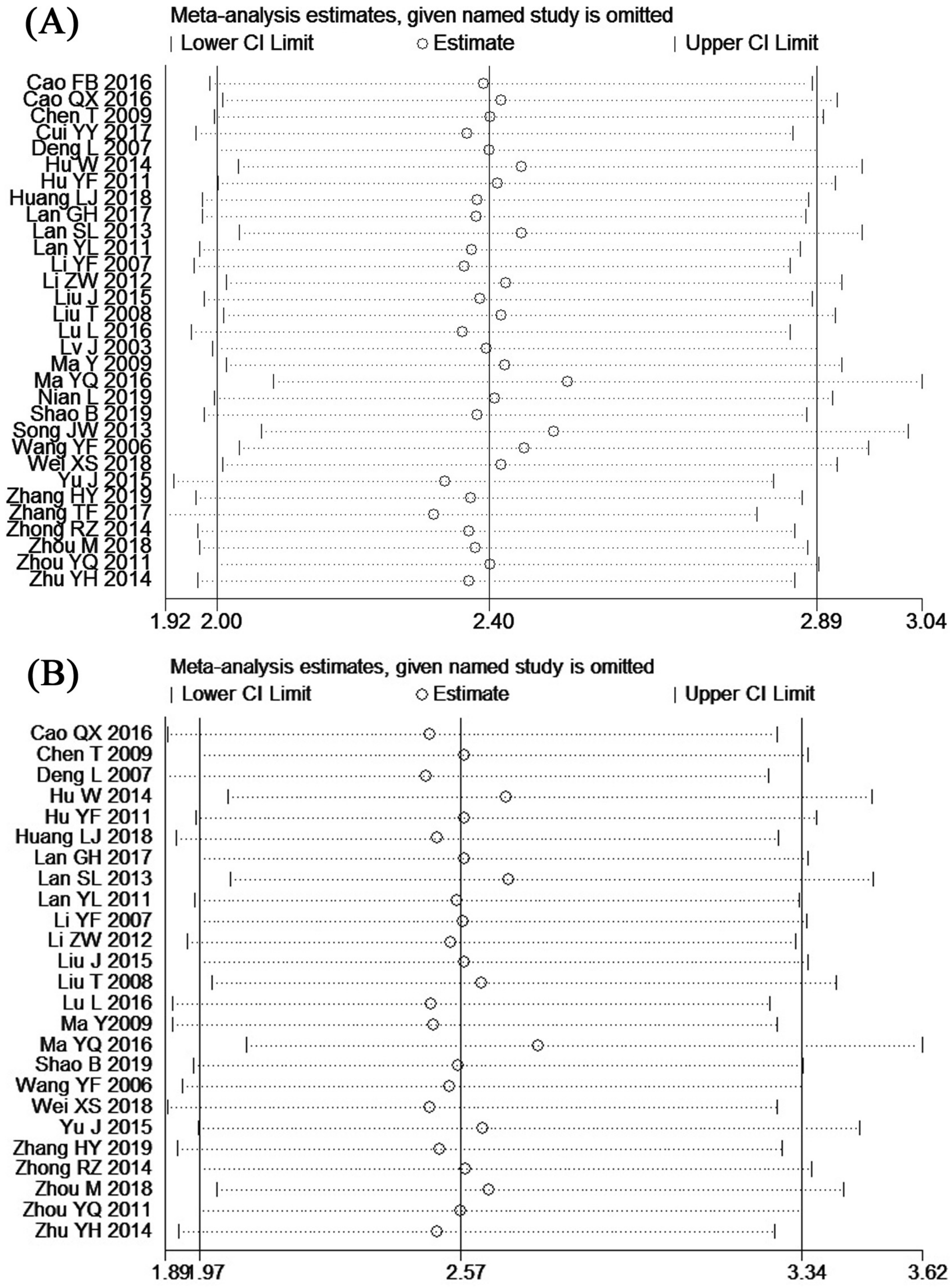
(A)
Meta-analysis estimates, given named study is omitted
Lower CI Limit
Estimate
Upper CI Limit
Cao FB 2016
Cao QX 2016
Chen T 2009
Cui YY 2017
Deng L 2007
Hu W 2014
Hu YF 2011
Huang LJ 2018
Lan GH 2017
Lan SL 2013
Lan YL 2011
Li YF 2007
Li ZW 2012
Liu J 2015
Liu T 2008
Lu L 2016
Lv J 2003
Ma Y 2009
Ma YQ 2016
Nian L 2019
Shao B 2019
Song JW 2013
Wang YF 2006
Wei XS 2018
Yu J 2015
Zhang HY 2019
Zhang TF 2017
Zhong RZ 2014
Zhou M 2018
Zhou YQ 2011
Zhu YH 2014
1.92
2.00
2.40
2.89
3.04
(B)
Meta-analysis estimates, given named study is omitted
Lower CI Limit
Estimate
Upper CI Limit
Cao QX 2016
Chen T 2009
Deng L 2007
Hu W 2014
Hu YF 2011
Huang LJ 2018
Lan GH 2017
Lan SL 2013
Lan YL 2011
Li YF 2007
Li ZW 2012
Liu J 2015
Liu T 2008
Lu L 2016
Ma Y2009
Ma YQ 2016
Shao B 2019
Wang YF 2006
Wei XS 2018
Yu J 2015
Zhang HY 2019
Zhong RZ 2014
Zhou M 2018
Zhou YQ 2011
Zhu YH 2014
1.89
1.97
2.57
3.34
3.62

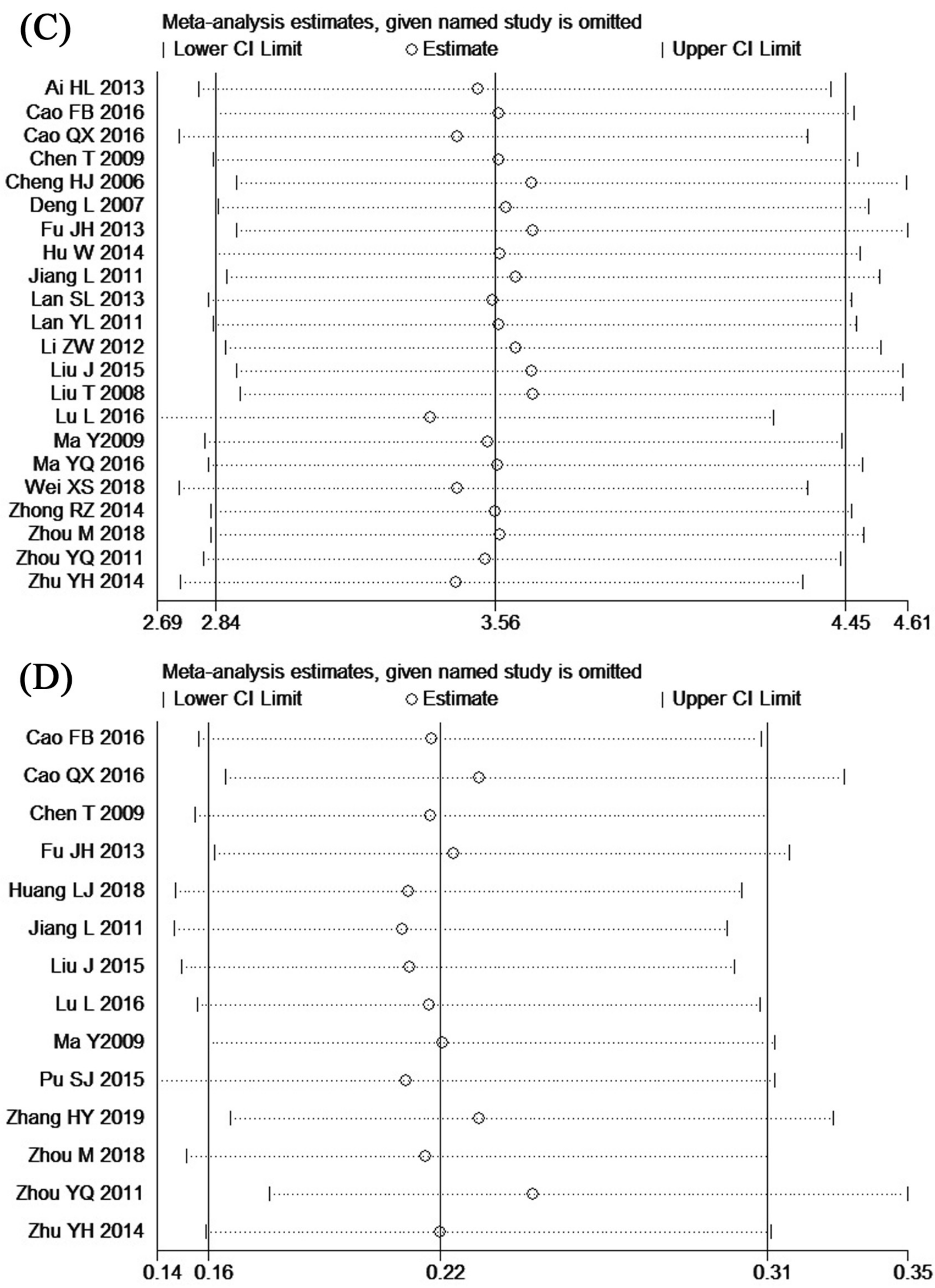
(C)
Meta-analysis estimates, given named study is omitted
Lower CI Limit
Estimate
Upper CI Limit
Ai HL 2013
Cao FB 2016
Cao QX 2016
Chen T 2009
Cheng HJ 2006
Deng L 2007
Fu JH 2013
Hu W 2014
Jiang L 2011
Lan SL 2013
Lan YL 2011
Li ZW 2012
Liu J 2015
Liu T 2008
Lu L 2016
Ma Y2009
Ma YQ 2016
Wei XS 2018
Zhong RZ 2014
Zhou M 2018
Zhou YQ 2011
Zhu YH 2014
2.69
2.84
3.56
4.45
4.61
(D)
Meta-analysis estimates, given named study is omitted
Lower CI Limit
Estimate
Upper CI Limit
Cao FB 2016
Cao QX 2016
Chen T 2009
Fu JH 2013
Huang LJ 2018
Jiang L 2011
Liu J 2015
Lu L 2016
Ma Y2009
Pu SJ 2015
Zhang HY 2019
Zhou M 2018
Zhou YQ 2011
Zhu YH 2014
0.14
0.16
0.22
0.31
0.35

(E)

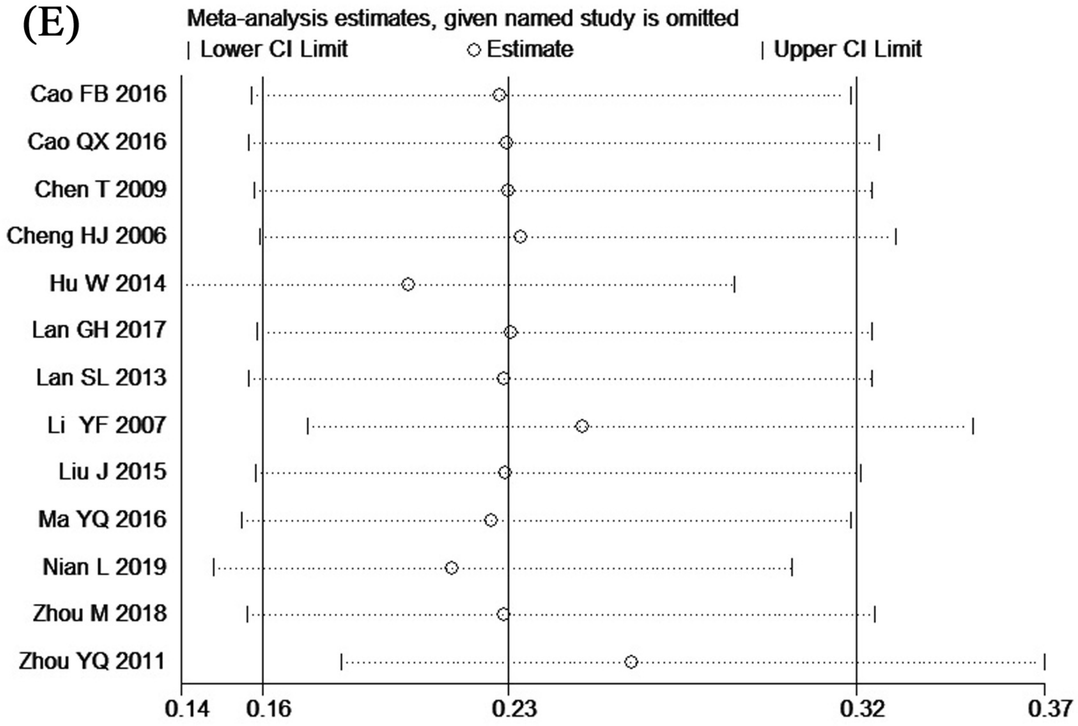
Meta-analysis estimates, given named study is omitted
Lower CI Limit
Estimate
Upper CI Limit
Cao FB 2016
Cao QX 2016
Chen T 2009
Cheng HJ 2006
Hu W 2014
Lan GH 2017
Lan SL 2013
Li YF 2007
Liu J 2015
Ma YQ 2016
Nian L 2019
Zhou M 2018
Zhou YQ 2011
0.14
0.16
0.23
0.32
0.37

(F)

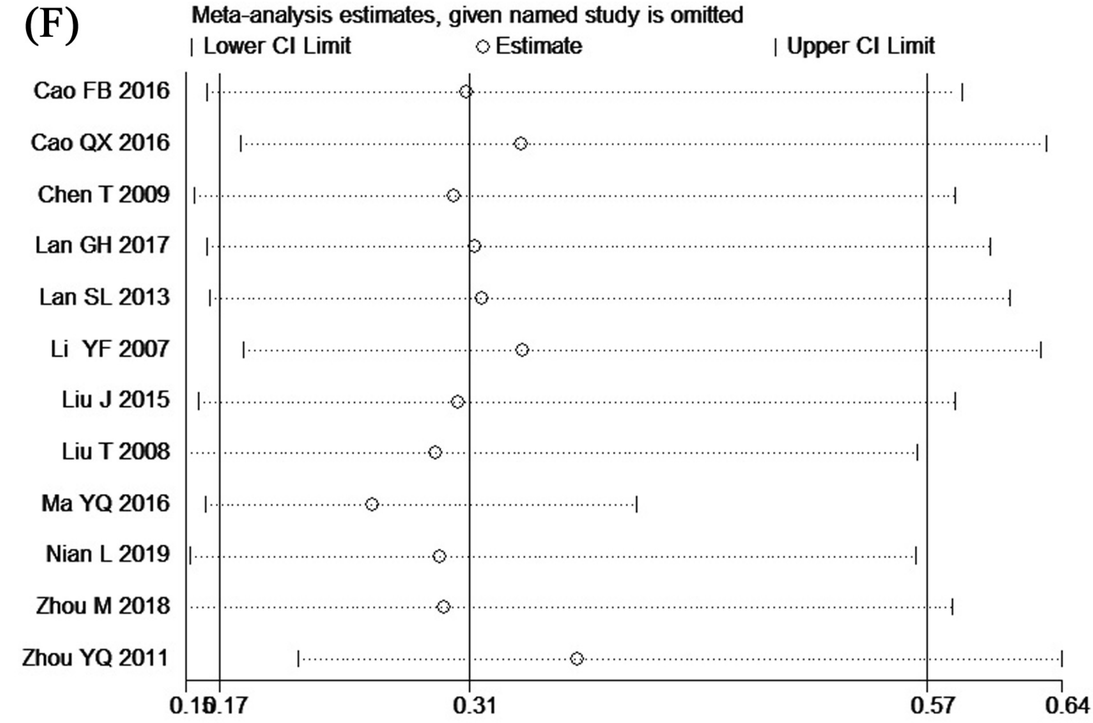
Meta-analysis estimates, given named study is omitted
Lower CI Limit
Estimate
Upper CI Limit
Cao FB 2016
Cao QX 2016
Chen T 2009
Lan GH 2017
Lan SL 2013
Li YF 2007
Liu J 2015
Liu T 2008
Ma YQ 2016
Nian L 2019
Zhou M 2018
Zhou YQ 2011
0.31
0.57
0.64

(G)

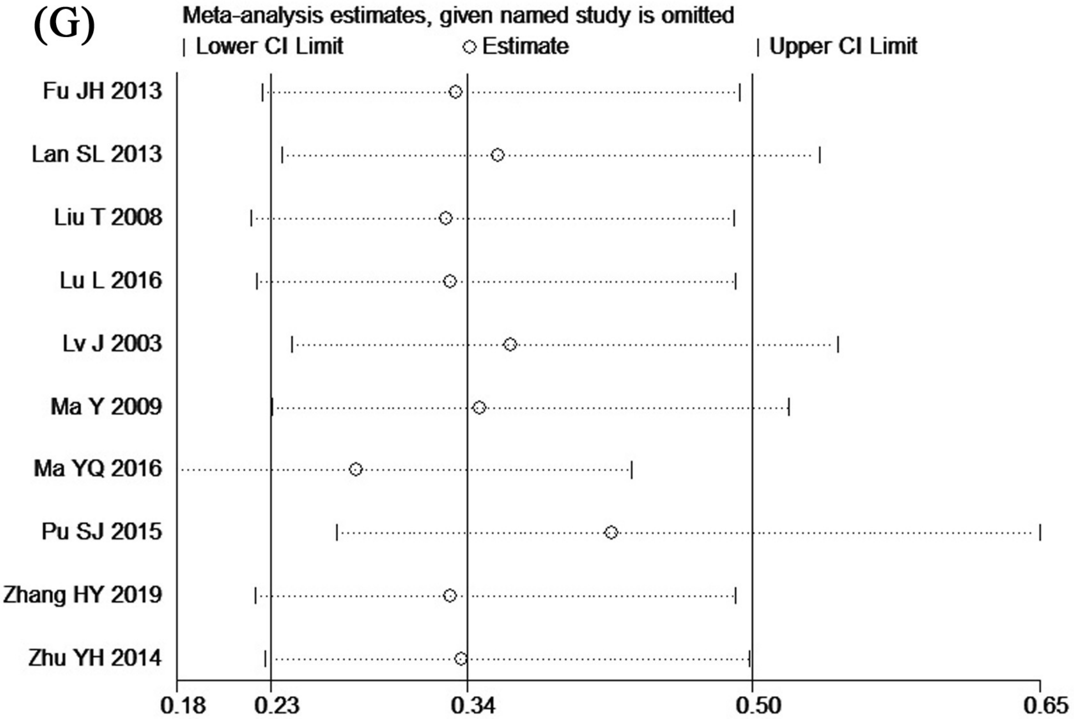
Meta-analysis estimates, given named study is omitted
Lower CI Limit
Estimate
Upper CI Limit
Fu JH 2013
Lan SL 2013
Liu T 2008
Lu L 2016
Lv J 2003
Ma Y 2009
Ma YQ 2016
Pu SJ 2015
Zhang HY 2019
Zhu YH 2014
0.18
0.23
0.34
0.50
0.65

Supplement Figure 3. Sensitivity analysis

(A) Sensitivity analysis for overall response rate; (B) Sensitivity analysis for disease control rate; (C) Sensitivity analysis for quality of life improved rate; (D) Sensitivity analysis for gastrointestinal adverse effects; (E) Sensitivity analysis for leukopenia; (F) Sensitivity analysis for thrombocytopenia; (G) Sensitivity analysis for hepatotoxicity. Parameters discussed less than 10 papers were not conducted sensitivity analyses.
